# Applications of Digital Health Technologies in Knee Osteoarthritis: Narrative Review

**DOI:** 10.2196/33489

**Published:** 2022-06-08

**Authors:** Nirali Shah, Kerry Costello, Akshat Mehta, Deepak Kumar

**Affiliations:** 1 Department of Physical Therapy and Athletic Training Boston University Boston, MA United States

**Keywords:** digital health, knee osteoarthritis, knee replacement, mobile health, telemedicine, mobile phone

## Abstract

**Background:**

With the increasing adoption of high-speed internet and mobile technologies by older adults, digital health is a promising modality to enhance clinical care for people with knee osteoarthritis (KOA), including those with knee replacement (KR).

**Objective:**

This study aimed to summarize the current use, cost-effectiveness, and patient and clinician perspectives of digital health for intervention delivery in KOA and KR.

**Methods:**

In this narrative review, search terms such as *mobile health*, *smartphone*, *mobile application*, *mobile technology*, *ehealth*, *text message*, *internet*, *knee osteoarthritis*, *total knee arthroplasty*, and *knee replacement* were used in the PubMed and Embase databases between October 2018 and February 2021. The search was limited to original articles published in the English language within the past 10 years. In total, 91 studies were included.

**Results:**

Digital health technologies such as websites, mobile apps, telephone calls, SMS text messaging, social media, videoconferencing, and custom multi-technology systems have been used to deliver interventions in KOA and KR populations. Overall, there was significant heterogeneity in the types and applications of digital health used in these populations. Digital patient education improved disease-related knowledge, especially when used as an adjunct to traditional methods of patient education for both KOA and KR. Digital health that incorporated person-specific motivational messages, biofeedback, or patient monitoring was more successful at improving physical activity than self-directed digital interventions for both KOA and KR. Many digital exercise interventions were found to be as effective as in-person physical therapy for people with KOA. Many digital exercise interventions for KR incorporated both in-person and web-based treatments (blended format), communication with clinicians, and multi-technology systems and were successful in improving knee range of motion and self-reported symptoms and reducing the length of hospital stays. All digital interventions that incorporated cognitive behavioral therapy or similar psychological interventions showed significant improvements in knee pain, function, and psychological health when compared with no treatment or traditional treatments for both KOA and KR. Although limited in number, studies have indicated that digital health may be cost-effective for these populations, especially when travel costs are considered. Finally, although patients with KOA and KR and clinicians had positive views on digital health, concerns related to privacy and security and concerns related to logistics and training were raised by patients and clinicians, respectively.

**Conclusions:**

For people with KOA and KR, many studies found digital health to be as effective as traditional treatments for patient education, physical activity, and exercise interventions. All digital interventions that incorporated cognitive behavioral therapy or similar psychological treatments were reported to result in significant improvements in patients with KOA and KR when compared with no treatment or traditional treatments. Overall, technologies that were blended and incorporated communication with clinicians, as well as biofeedback or patient monitoring, showed favorable outcomes.

## Introduction

Digital health can be broadly defined as the use of technologies such as websites, mobile phones, wearable devices, and telemedicine for the diagnosis, treatment, prevention, and maintenance of health [1]. Digital health has been increasingly used for remote and personalized care across a range of health conditions, and the COVID-19 pandemic has further highlighted the need for and accelerated the adoption of these technologies [2]. With the increasing use of the internet and mobile computing devices in older adults [3-5], digital health holds promise for clinical and research applications in people with knee osteoarthritis (KOA) [6].

The core recommendations for KOA management include patient education, self-management, and exercise [7-11]. However, current treatment approaches are largely inconsistent with the guidelines [12,13]. Barriers to the implementation of clinical practice guidelines in osteoarthritis include limited access to health care settings, lack of knowledge of treatment approaches and guidelines, psychological barriers (eg, poor self-efficacy), and system-related factors (eg, limited health care provider time) [14,15]. Digital health may help address many of these barriers and increase the uptake of clinical practice guidelines, for example, by improving access to care and information, delivery of behavioral interventions, and remote patient monitoring.

Prior reviews on digital health for the management of KOA were mostly systematic reviews [16-21]. These systematic reviews focused on one type of digital health (eg, telerehabilitation [16,17] or mobile health technology [18,20]) or on one rehabilitation goal (self-management [21]) or only included populations with knee replacement (KR) surgeries [16,17,19]. Although systematic reviews are rigorous, they tend to have a narrow scope because of the focus on evidence related to the effectiveness of interventions [22]. Currently, a comprehensive overview with a wider focus on the various digital health technologies used for the management of KOA is lacking in the literature. Such a review is needed to identify what has been accomplished in the field of digital health, thus allowing researchers and clinicians to build on previously published research. Thus, the objective of this narrative review was to summarize the current state of digital health in KOA and provide an overview of the cost-effectiveness and patient and clinician perspectives related to digital health in these populations.

## Methods

A literature search was conducted in 2 databases, PubMed and Embase, in October 2018, November 2019, and February 2021. The keywords used for the search at all 3 time points were as follows: (*mobile health* OR *mobile phone* OR *smartphone* OR *mobile application* OR *mobile* *technology* OR *ehealth* OR text message* OR *mhealth* OR *internet* OR *web based* OR *social media* OR *Facebook* OR *YouTube* OR *Twitter*) AND (*osteoarthriti*s OR *TKA* OR *total knee arthroplasty* OR *total knee replacement*).

The inclusion criteria were (1) original studies published in the English language, (2) studies published in the past 10 years, and (3) technologies used for rehabilitation of KOA or KR. Studies that investigated the use of technology for diagnosis, decision aid, informed consent, or movement assessments were excluded from this review. Furthermore, duplicates, conference abstracts, protocol papers, and previously published reviews, including systematic reviews, were excluded. One of the researchers (NS) initially screened the titles of the studies in the search results against the aforementioned inclusion and exclusion criteria, removing studies that were not relevant to the review. The remaining studies were reviewed by 3 researchers (NS, KEC, and DK) who read the abstracts of each study to determine whether they should be included in the review. For the included studies, one of the authors (NS) extracted pertinent information as applicable, including objective, design, intervention characteristics, outcomes and findings, and limitations. After reviewing this information, we grouped the studies based on the applications of digital health to organize this review for the readers. We grouped the studies into digital health for delivering patient education, physical activity, exercise (asynchronous and synchronous exercise delivery), and psychological treatments such as cognitive behavioral therapy (CBT) or pain coping skills training (PCST) in the KOA and KR populations. We also discuss the findings related to cost-effectiveness and patient and clinician perspectives on digital health.

## Results

**Figure 1:**
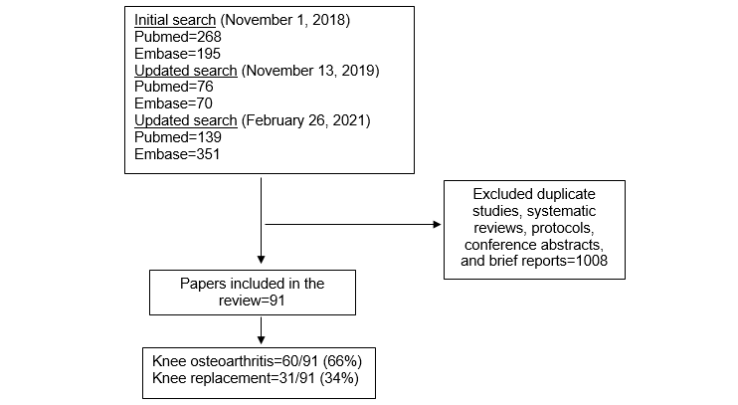
Flow diagram of the search process.

After a careful review process, 91 studies were included in this review (Figure 1). Of the 91 studies included in this review, 60 (66%) were from KOA populations and 31 (34%) were from KR populations.

### Digital Health for Patient Education

#### Overview

This section includes interventions that delivered patient education to individuals with KOA or KR to improve disease-related knowledge and symptoms related to osteoarthritis. We defined patient education as information on a health condition, its treatment, and related self-management techniques [7]. This section also includes studies that have investigated the educational quality of content on osteoarthritis-related websites or videos on YouTube. Although a brief overview of the studies included in this section is shown in Table 1, a detailed description of the studies and features of technology used in the studies included in this section is presented in Table S1 in Multimedia Appendix 1 [23-35].

**Table 1:** Digital health for patient education in people with KOA^a^ and KR^b^.

Study	Population	Design	Intervention			Comparator			Primary outcome findings
			Description	Sample size	Description		Sample size		
Brosseau et al [28]	Self-reported osteoarthritis or RA^c^	Pre or post	Social media (Facebook)	41	N/A^d^		N/A	Improvements in disease-related knowledge from baseline	
Umapathy et al [24]	Knee or hip osteoarthritis	Pre or post	Access to website-based education and use of the website	104	Access to website-based education but no use of the website		91	Significant improvements in the Osteoarthritis Quality Indicator measures for users of the website vs no significant improvement for nonusers	
Timmers et al [23]	Knee pain	RCT^e^	Phone app providing daily patient education	91	Information offered during medical consultation		122	Disease-related knowledge was 52% higher in the intervention group	
Wang et al [25]	Knee or hip osteoarthritis	Quasi-experimental study	Users of the updated version of My Joint Pain for education	35	Nonusers		87	No significant difference in the Health Evaluation Impact Questionnaire scores between users and nonusers of the website	
Fraval et al [26]	Presurgery (KR or HR^f^)	RCT	Website+discussion with surgeon	103	Discussion with surgeon		108	Improvements in disease-related knowledge but not anxiety scores in the intervention vs comparator	
Campbell et al [27]	Postsurgery (KR or HR)	RCT	SMS text messaging bot+traditional education	76	Traditional education		83	Improvements in exercise adherence in the intervention vs comparator	
Timmers et al [35]	Postsurgery (KR)	RCT	Phone app providing specific education at specific times from date of discharge	114	Phone app providing standard education biweekly		99	The intervention group had improvements in pain on NRS^g^ at rest, at night, and during activity vs the comparator at 4 weeks after discharge	
Meldrum et al [29]	Knee pain	Qualitative content analysis	Comments on videos related to knee pain on YouTube	3537 (comments) and 58 (videos)	N/A		N/A	Comments included soliciting advice for knee pain (19%), appreciation for others’ inputs (17%), and asking questions regarding videos (15%)	
Barrow et al [30]	Osteoarthritis	Cross-sectional survey	Websites providing educational content for patients with osteoarthritis	50	N/A		N/A	68% of the websites scored more than half of the maximum available quality score	
Murray et al [32]	Osteoarthritis	Readability and quality assessment	Websites on osteoarthritis	37	N/A		N/A	Readability ranged from 8th- to 12th-grade reading level, and the quality of web-based osteoarthritis information was rated as “poor” to “fair”	
Chapman et al [31]	Osteoarthritis	Nonexperimental, descriptive, internet-based study	Websites on self-management in knee, hip, hand osteoarthritis	49	N/A		N/A	Reading grade levels ranged from 6 to 15	
Wong et al [34]	Osteoarthritis	Quality assessment	Videos on KOA and KR on YouTube	56	N/A		N/A	Approximately 65% of videos had poor educational quality, 30% had acceptable educational quality, and <10% had good educational quality	
Bahadori et al [33]	KR	Readability assessment	Information on KR apps	15	N/A		N/A	Only one app was found to be “easy to read”	

^a^KOA: knee osteoarthritis.

^b^KR: knee replacement.

^c^RA: rheumatoid arthritis.

^d^N/A: not applicable.

^e^RCT: randomized controlled trial.

^f^HR: hip replacement.

^g^NRS: Numeric Pain Rating Scale.

#### Patient Education for People With KOA: Facebook, Mobile App, and Website

Approximately 2% (2/91) of studies, one single-arm study and a randomized controlled trial (RCT), found significant improvements in disease-related knowledge in people with KOA with the use of a Facebook group page (*People getting a grip on arthritis II*) [28] and with a mobile app (*Patient Journey App*) compared with education via medical consultation [23]. In contrast, health education via an open-access website (*My Joint Pain*) [24,25] did not result in significant improvements in health education outcomes such as the Health Evaluation Impact Questionnaire and the Osteoarthritis Quality Indicator, even with the updated version of the website [25]. Although these findings might suggest that osteoarthritis education via an open-access website [24,25] does not improve disease-related knowledge compared with a mobile app [23] or Facebook group page [28], it is important to consider that assessment of disease-related knowledge with the open-access website was done much later (12 and 24 months) than assessments of disease-related knowledge with the mobile app (7 days) and Facebook group page (3 months) [23-25,28]. Second, the studies with open-access websites reported higher attrition rates than the studies with mobile apps and Facebook group pages (29% and 30% vs 22% and 16%) [23-25,28]. Furthermore, although the open-access website *My Joint Pain* [24,25] allowed users to access the website at their convenience, both the mobile app [23] and Facebook group page [28] interventions improved engagement with push notifications and reminders. Notably, the mobile app also included features such as web-based quizzes [24], and the Facebook group page [28] incorporated peer support by allowing users to comment on and share their experiences with the health education videos. Collectively, this evidence suggests that digital patient education (mobile apps and Facebook group pages) improves disease-related knowledge at shorter follow-up periods and that it might be helpful to include features such as feedback, push notifications, and reminders in a digital intervention for people with KOA.

#### Patient Education for People With KR: Website, Text Messaging, and Mobile Apps

Fraval et al [26] reported greater improvements in knowledge (regarding orthopedic surgery) in people who received website-based disease-related education along with a surgical consultation than in people who received the surgical consultation alone. Similarly, those who received (automated) encouraging SMS text messages and personalized video messages from their surgeons regarding recovery along with traditional perioperative education spent more time participating in home exercises than participants who only received perioperative education (mean difference 8.6 minutes; *P*<.001) [27]. In terms of postoperative pain, Timmers et al [35] found statistically significant but clinically nonsignificant, improvements in pain outcomes in people who used a mobile app delivering specific information related to the individual’s recovery compared with people who received basic unstructured information biweekly through the app [35]. Timmers et al [35] also found that using push notifications to alert users of new information resulted in the increased use of the app by the user (26 times per user). Overall, these studies suggest that education via different digital modes (ie, websites, SMS text messages, or mobile apps) improves surgery-related knowledge, time spent performing exercises, and pain outcomes in people undergoing KR. Moreover, similar to populations with KOA, it might be beneficial to include features such as push notifications to improve engagement in digital interventions for individuals with KR.

#### Educational Quality of Web-Based Information on KOA or KR: Websites, Mobile Apps, and YouTube Videos

For KOA, information on websites was investigated. Although the educational quality of information related to KOA has improved recently, there is still poor readability, substantial variability, and inconsistencies in the information available on websites [29-32]. For KR, the information on mobile apps was investigated; however, no app that provided information related to KR met the recommended readability levels (the one app that was found *easy to read* provided information on hip replacement surgeries) [33]. Similar to websites and mobile apps, the educational quality of information related to KOA and KR on YouTube has also been found to be of poor quality [34]. Despite issues with educational quality, analysis of the comments section on YouTube videos on knee pain management revealed that people with knee pain were comfortable sharing experiences and seeking advice on knee pain from other people on YouTube [29]. Therefore, although peer support via digital health can serve as a useful and informative tool for patients, the current educational and readability quality of osteoarthritis-related information needs improvement.

### Digital Health for Physical Activity Interventions

#### Overview

This section includes interventions that were delivered with the purpose of improving physical activity (ie, step count, mobility, and time spent inactive in people with KOA; Table 2). A detailed description of the studies and technology used in the papers in this section is shown in Table S2 in Multimedia Appendix 1 [36-41].

**Table 2:** Digital health for PA^a^ interventions in people with knee osteoarthritis.

Study	Population	Design	Intervention			Comparator or comparators			Primary outcome findings
			Description	Sample size	Description		Sample size		
Bossen et al [38]	Knee or hip osteoarthritis	Pre or post	Join2Move (fully automated web-based PA program)	20	N/A^b^		N/A	No improvements in PA or self-perceived effect	
Li et al [41]	Knee osteoarthritis	RCT^c^	Group in-person education+ activity monitor+ telephone counseling	17	Same intervention delayed by 1 month		17	Greater improvement in moderate to vigorous PA in the intervention vs comparator	
Skrepnik et al [36]	Knee osteoarthritis treated with Hylan G-F 20	RCT	Hyaluronic acid injection+unblinded activity monitor phone app	107	Hyaluronic acid injection+blinded activity monitor		104	Improvements in mobility in the intervention vs comparator	
Bartholdy et al [37]	Knee osteoarthritis	RCT	Motivational SMS text messaging related to PA	19	No treatment		19	No difference between groups for time spent physically inactive	
Zaslavsky et al [39]	Osteoarthritis	Pre or post	Activity monitor, motivational SMS text messaging, telephone coaching, and phone app for feedback	24	N/A^b^		N/A	Improvements in sleep but not PA from baseline	
Allen et al [40]	Knee or hip osteoarthritis	Pre or post	PA screening, coaching phone calls, emails, and phone follow-up	67	N/A^b^		N/A	No difference in improvement in minutes of moderate to vigorous PA	

^a^PA: physical activity.

^b^N/A: not applicable.

^c^RCT: randomized controlled trial.

#### Digital Health for Physical Activity in People With KOA: Mobile App, Text Messaging, Multi-Technology, and Website

Digital physical activity interventions for people with KOA were delivered via website programs (1/91, 1%), telephone calls or SMS text messaging (1/91, 1%), mobile apps with or without activity monitors (1/91, 1%), or a combination of these technologies (3/91, 3%). Although 4% (4/91) of these interventions were self-directed or self-paced [36-39], 2% (2/91) of physical activity interventions included calls with a personal coach [40] and physical therapist [41] for individualized goal setting.

In an RCT, Skrepnik et al [36] reported greater improvements in daily step counts in adults with KOA after 90 days of using an activity monitor with visible feedback and access to a mobile app (*OA GO*) than in those who used a blinded activity monitor, despite regular follow-ups with care providers for both groups. The mobile app *OA GO* in this study provided motivational SMS text messages (on pain and mood monitoring) along with feedback, progress reports, and monthly trends related to physical activity from the activity monitor [36]. However, when SMS text messages related to generic physical activity advice were given to people with KOA, the improvements in physical activity and the time spent inactive were nonsignificant compared with those who received no treatment [38]. The findings of these studies indicated that visible biofeedback and user-relevant content with motivational interviewing principles might be more effective in improving physical activity than general physical activity advice. These findings were confirmed by Li et al [41] in a delayed-control design, preliminary RCT, where an initial in-person education session, activity monitor, and weekly telephone coaching provided by physical therapy (PT) were successful in improving physical activity and reducing sedentary behavior in people with KOA, suggesting that a blended format (a combination of in-person and digital) might also be beneficial for favorable results. However, when a single-arm pilot study used a mobile app for biofeedback from an activity monitor, personalized weekly SMS text messages, and motivational interviewing via 3 phone calls, they found no significant improvement in the overall step counts at 14 or 19 weeks [39]. As the participants in the study discussed valuing the person-specific messages during the exit interviews, the authors speculated that the nonsignificant findings might be related to the insufficient frequency of SMS text messages (weekly) during the study [39].

Similar nonsignificant improvements in physical activity were reported in 2% (2/91) of single-arm pilot studies that used a website (*Join2Move*) and a multi-technology web-based intervention (osteoarthritis physical care pathway). The website (*Join2Move*) was self-paced, fully automated, and provided weekly physical activity assignments based on the goals and a self-test of recreational activities selected by the user [38]. The use of a self-directed intervention (*Join2Move*) with minimum personal contact resulted in a high attrition rate, with only 55% of participants completing at least 75% of the program, potentially resulting in nonsignificant improvements in physical activity [38]. In contrast, the osteoarthritis physical care pathway intervention used the website and telephone calls for 4 phases (ie, physical activity screening, brief coaching calls for goal setting based on motivational interviewing principles, access to community and local resources to support physical activity, and follow-up coaching calls) [40]. Although this intervention included person-specific information using motivational interviewing, it did not include visible biofeedback or physical activity self-monitoring, which might have resulted in nonsignificant results. Interestingly, although 5% (5/91) of studies in this section found no significant changes in physical activity [36-40], 2% (2/91) of studies observed statistically significant but clinically nonsignificant improvements in secondary outcome measures of sleep [39] and pain and function subscales on the Western Ontario and McMaster Universities Osteoarthritis Index (WOMAC) [40].

#### Digital Health for Physical Activity in People With Knee KR

No studies investigating physical activity interventions in people with KR were identified.

### Digital Health for Exercise Interventions

#### Overview

Exercise remains the most effective nonpharmacologic intervention for KOA [7,9]. This section includes interventions that delivered exercises (ie, a structured program for the purpose of improving osteoarthritis-related symptoms) to people with KOA and KR (Table 3). A detailed description of the studies and technology used in the studies in this section is shown in Table S3 in Multimedia Appendix 1 [6,42-53].

**Table 3:** Self-directed or asynchronous digital exercise interventions.

Study	Population	Design	Intervention		Comparator or comparators			Primary outcome findings
			Description	Sample size	Description	Sample size		
Dahlberg et al [44]	Knee or hip osteoarthritis	Pre or post	Joint Academy (website with videos on education and exercise and asynchronous chat support from PT^a^)	53	N/A^b^	N/A	68% (16/53) of responders defined by individual improvement of >1.5 on the NRS^c^ pain score	
Nero et al [45]	Knee or hip osteoarthritis	Observational and quasi-experimental	Joint Academy (website with videos on education and exercise and asynchronous chat support from PT)	350	Published data from in-person PT	—^d^	Significant improvements in NRS pain score or function on 30-second chair stand test	
Allen et al [52]	Knee osteoarthritis	RCT^e^	IBET^f^ (website with tailored exercise, exercise videos, automated reminders, and progress tracking)	140	In-person PT and waitlist control	140 (in-person) and 70 (waitlist)	No difference between groups for improvements in WOMAC^g^ score	
Pignato et al [43]	Knee osteoarthritis	Secondary analysis from an RCT [52]	Website	124	In-person PT	135	More PT visits resulted in greater improvement in WOMAC scores	
Nelligan et al [42]	Knee osteoarthritis	Participants and assessors blinded RCT	My Knee Exercise website with education+prescription for a 24-week knee strengthening regimen+ automated personalized SMS text messages	103	Access to My Knee Exercise website with education+automated SMS text messages without specific information on exercises	103	Greater improvements in overall knee pain and WOMAC function in the intervention vs comparator	
Dahlberg et al [6]	Knee or hip osteoarthritis	Longitudinal cohort study	Joint Academy website with videos on education and exercise and asynchronous chat support from PT	499	N/A	N/A	Improvement in monthly NRS pain score and physical function on 30-second chair stand test at week 12	
Gohir et al [53]	Knee osteoarthritis	RCT	Joint Academy website	48	Usual care delivered by a general practitioner or physical therapist	57	Greater improvements in NRS pain score in the intervention vs comparator at 6 weeks	
Piqueras et al [51]	Post-KR^h^	RCT	Asynchronous platform with inertial sensors to measure movement, avatar-based exercise, and web portal for PT	90	In-person PT	91	No difference in knee flexion and extension after the intervention between groups	
Bini et al [50]	Post-KR	RCT	Phone app with videos prescribed by PT	14	In-person outpatient PT	15	No difference between groups for VAS^i^, Veterans RAND 12-item health survey mental component and physical component scores, and KOOS^j^	
Chughtai et al [46]	Pre-KR	Pre or post study	Mobile app “PreHab” with prehabilitation program before TKA^k^	114	Nonusers	362	Shorter length of stay in the hospital and more favorable discharge disposition status in those who used the app	
Fleischman et al [49]	Post-KR	Randomized noninferiority trial	Inpatient PT until hospital discharge+web-based unsupervised PT with patient monitoring and communication portal	96	Inpatient PT until hospital discharge+printed PT manual and in-person PT	96 (inpatient) and 97 (in-person)	No difference in change in knee flexion in intervention and comparator at 4-6 weeks or 6-months postop	
Klement et al [48]	Post-KR	Retrospective intervention	Web-based self-directed PT—automated emails with exercises	296	In-Person PT+web-based self-directed PT	101	Greater difference in knee flexion, SF-12^l^ physical scores, and KOOS pain but not knee extension or SF-12 mental scores in the intervention vs comparator	
Ramkumar et al [47]	Pre-KR	Pre or post	Knee sleeve with inertial sensors+phone app	25	N/A	N/A	Improvements in mobility but not knee flexion or KOOS scores at 3 months after operation	

^a^PT: physical therapy.

^b^N/A: not applicable.

^c^NRS: Numeric Pain Rating Scale.

^d^Not available.

^e^RCT: randomized controlled trial.

^f^IBET: Internet-Based Exercise Therapy.

^g^WOMAC: Western Ontario and McMaster Universities Osteoarthritis Index.

^h^KR: knee replacement.

^i^VAS: Visual Analog Scale.

^j^KOOS: Knee Osteoarthritis Outcome Score.

^k^TKA: total knee arthroplasty.

^l^SF-12: Short Form-12.

#### Self-directed and Asynchronous Digital Exercise Interventions for People With KOA: Websites and Mobile App

This section includes exercise interventions that were not delivered by a physical therapist in real time. Specifically, the interventions that were self-directed or monitored through asynchronous communication with a physical therapist (communication portal or chat feature on a website: 4/91, 4%; videos uploaded on a mobile app: 1/91, 1%) are included in this section.

In a parallel superiority RCT, Nelligan et al [41] found that people with KOA who received additional strength training, personalized SMS text messages, and guidance to improve physical activity along with disease-related education (*My Knee Exercise* website) showed greater improvements in pain on the Numeric Pain Rating Scale and function on the WOMAC than people who only received access to the disease-related education on the website (*My Knee Education*). Moreover, the within-group improvements in pain on the Numeric Pain Rating Scale and function on the WOMAC in the *My Knee Exercise* group had large effect sizes and exceeded the minimal clinically important difference (MCID) in the study [54,42]. In another RCT, Allen et al [40,52] (Physical Therapy versus Internet-Based Exercise Training [PATH-IN] trial) compared an unsupervised website exercise program called Internet-Based Exercise Therapy (IBET) with in-person PT and waitlist controls. Interestingly, the study found that IBET was noninferior to in-person PT in improvements on the WOMAC and that both IBET and in-person PT were not superior to the waitlist at the 4 or 12 months follow-up [52]. However, the within-group improvements in all 3 groups were above the minimal clinically important changes (>1.33 points) [55] at 4 and 12 months but had small effect sizes [52]. Notably, IBET [52] had more features and flexibility (tailored exercise videos, exercise progressions, automated reminders, and progress tracking) than *My Knee Exercise* (education, a prescription for 24-week strengthening exercises, and personalized SMS text messages) [42]. The authors of the PATH-IN trial speculated that greater doses in both intervention groups and greater engagement in the IBET group may be needed to determine efficacy. However, secondary analyses from the PATH-IN trial did not show an association between adherence to IBET and changes in outcomes, and interestingly, no participant characteristics were related to adherence to IBET [43].

Approximately 2% (2/91) of single-arm studies investigated a 6-week website program called the *Joint Academy*, a program comprising short educational lectures, daily exercise videos, and asynchronous chats with physical therapists [44,45]. These studies reported statistically significant but clinically not significant [54,56,57] improvements in pain [44,45], function [45], and walking difficulty [45]. Similarly, when a mobile app version of the *Joint Academy* was used, there were statistically significant and clinically nonsignificant improvements in pain and function at 6 weeks when compared with usual care [53]. However, a longitudinal cohort study used data from a self-management program registry and found that 72% and 67% of participants who used *Joint Academy* achieved the MCID for pain [54,57] at longer follow-up periods of 24 and 48 weeks, respectively, therefore suggesting that longer digital health interventions may be required for clinical benefits [6]. Moreover, given that these digital health interventions were not directly compared with in-person PT, it is unclear whether they are superior or similarly effective compared with in-person PT.

#### Self-directed and Asynchronous Digital Exercise Interventions for People With KR: Multi-Technology and Websites

For people undergoing KR, self-directed exercise interventions were provided using multi-technology (2/91, 2%) systems [46,47]. Ramkumar et al [47] used a knee sleeve with inertial motion sensors and a mobile app, and Chughtai et al [46] used a web or phone-based platform with a daily activity checklist, exercise instructions, nutritional advice, education, mindfulness, and other components. Both studies reported significant within-group improvements in mobility, symptoms, length of hospital stay, and other outcomes with their multi-technology systems [46,47]. For individuals after KR, 2% (2/91) of RCTs investigated the use of self-directed website exercise interventions [48,49]. Fleischman et al [49] found similar improvements in knee range of motion and self-reported symptoms on the Knee Osteoarthritis Outcome Score in those who received the website intervention and in those who received in-person PT at short (4-6 weeks) and long (6 months) follow-up periods. Similarly, Klement et al [48] found that 65.9% of participants who received their self-directed website intervention did not require in-person PT 2 weeks after the operation. The improvements with these website interventions may be because of their various features such as weekly exercise programs with video demonstrations [48,49], patient monitoring [49], and a communication portal for asynchronous conversation with the physical therapist [49]. Similarly, telerehabilitation exercise programs that allowed communication with a physical therapist (telephone) and patient monitoring via asynchronous video uploads [50] and sensor-based feedback [51] elicited similar improvements in knee range of motion and self-reported symptoms as in-person PT at early (10 days) and later (3 months) follow-up periods [50,51]. Taken together, multi-technology self-directed exercise interventions and websites that allow biofeedback, patient monitoring or communication with clinicians have been successful in eliciting positive outcomes such as range of motion and self-reported symptoms in people with KR.

#### Directly Supervised Exercise Interventions for Populations With KOA: Blended and Telephone-Based

This section includes exercise interventions that were directly delivered by a clinician, generally physical therapists, in real time (Table 4). A detailed description of the studies and the technology used in the studies in this section is presented in Table S4 in Multimedia Appendix 1 [58-74]. The exercise interventions in this section were provided in blended formats (ie, a combination of in-person PT and digital strategies [58,59,73] or over the phone) [60,61] or via real-time videoconferencing software.

**Table 4:** Digital health for directly supervised exercise interventions.

Study	Population	Design	Intervention			Comparator or comparators			Primary outcome findings
			Description	Sample size	Description		Sample size		
Cuperus et al [73]	Generalized osteoarthritis	RCT^a^	2 in-person group sessions+telephone monitoring by a nurse	77	Multidisciplinary in-person group intervention led by PT^b^		81	No difference in daily function on Health Assessment Questionnaire Disability Index between groups	
Bennell et al [70]	Inactive adults with knee osteoarthritis	RCT	In-person PT+telephone coaching	84	In-person PT		84	Greater improvements in the NRS^c^ pain score and the WOMAC^d^ function in the intervention vs comparator	
Kloek et al [59]	Knee or hip osteoarthritis	Cluster RCT	Website+in-person PT	109	Usual in-person PT		99	No difference between groups for KOOS^e^, timed up and go, and subjective and objective physical activity	
De Vries et al [62]	Knee or hip osteoarthritis	Mixed methods study embedded within an RCT [59]	Web-based component of e-exercise used by Kloek et al [59]	Quantitative analysis=90; qualitative analysis=10	N/A^f^		N/A	Adherence was highest for participants with middle education, 1- to 5-year osteoarthritis duration, and participants who were recruited by physical therapists	
Chen et al [58]	Knee osteoarthritis	Quasi-experimental study	Blended intervention: in-person group PT+home exercises, exercise diary, and telephone check-in calls	84	In-person group health education sessions and telephone check-in calls		87	Greater improvements for WOMAC pain and joint stiffness on a Likert scale in the intervention vs comparator	
Baker et al [60]	Knee osteoarthritis	Single-blind parallel-arm RCT	BOOST-TLC^g^ (motivational behavior change telephone calls+monthly automated phone reminder messages to exercise)	52	Monthly automated phone reminder messages to exercise		52	No difference between groups in adherence	
Doiron-Cadrin et al [63]	Pre-KR^h^ and HR^i^	RCT	Real-time videoconferencing	12	In-person outpatient PT and usual care		12 (in-person) and 11 (usual care)	High compliance and satisfaction with the teleprehabilitation program	
Hinman et al [61]	Knee osteoarthritis	Participant and assessor–blinded RCT	5-10 calls from a physical therapist for exercise advice and prescription+information folder+exercise bands+access to website for exercise videos+≥1 call from a nurse for self-management advice	87	≥1 telephone call from a nurse for self-management advice		88	Improvements in function but not pain in the intervention vs comparator	
Lawford et al [71]	Knee osteoarthritis	Exploratory trial using data from the intervention arm of RCT [61]	5-10 calls from a physical therapist for exercise advice and prescription+information folder+exercise bands+access to website for exercise videos+≥1 call from a nurse for self-management advice	87	N/A		N/A	Weak association between therapeutic alliance and improvements in knee pain, self-efficacy, function, quality of life, adherence, and physical activity	
Russell et al [72]	Post-KR	RCT	Computer-based system with real-time videoconferencing, measurement tools, and video capture	31	In-person outpatient PT		34	No difference between groups for improvement in WOMAC scores	
Tousignant et al [65]	Post-KR	RCT	Custom hardware with videoconferencing and remote-controlled cameras	24	In-person PT		24	No significant difference between groups for knee extension and WOMAC total score	
Moffet et al [64]	Post-KR	RCT	Custom hardware with videoconferencing and remote-controlled cameras	104	In-person home-based PT		101	No difference in WOMAC score between groups	
Correia et al [69]	Post-KR	RCT	Platform with inertial sensors, phone app, and web portal for PT+2 home visits and telephone support by PT	30	In-person home-based PT		29	Greater improvement in the intervention vs comparator for timed up and go scores at 8 weeks	
Correia et al [68]	Post-KR	RCT	Platform with inertial sensors, phone app, and web portal for PT+2 home visits and telephone support by PT	30	In-person home-based PT		29	Greater improvement in the intervention vs comparator for timed up and go scores at 6 months	
Bell et al [66]	Post-KR	Pilot RCT	In-person PT+interACTION (monitoring remote rehabilitation platform with portable IMUs^j^+mobile app with back end clinician portal)	13	In-person PT+unsupervised home exercise program		12	No difference in value (change in activities of daily living scale and total cost) between groups	
Chughtai et al [67]	Post-KR	Pre or post	3D motion-tracking cameras, exercise avatar, clinician monitoring, outcome reporting, and communication with a clinician—TKA^k^ and UKA^l^	18 (TKA) and 139 (UKA)	N/A		N/A	Improvements in Knee Society Scores, WOMAC scores, and Boston University Activity Measure for Post-Acute Care scores	
El Ashmawy et al [74]	Post-KR or HR	Retrospective study	Remote joint replacement clinic follow-up at 1-year, 7-years, and every 3-years after in-person consultations at 2 weeks and 6-weeks	1749	N/A		N/A	92% response rate, 87% completed the outcome forms and radiographs, 7% required further in-person appointments, and 89% satisfaction; 1 web-based appointment cost £79 (US $99), with estimated savings of £42,644 (US $53,439.93) per year^m^	

^a^RCT: randomized controlled trial.

^b^PT: physical therapy.

^c^NRS: Numeric Pain Rating Scale.

^d^WOMAC: Western Ontario and McMaster Universities Osteoarthritis Index.

^e^KOOS: The Knee Osteoarthritis Outcome Score.

^f^N/A: not applicable.

^g^BOOST-TLC: Boston Overcoming Osteoarthritis through Strength Training Telephone-linked Communication.

^h^KR: knee replacement.

^i^HR: hip replacement.

^j^IMU: inertial motion sensor.

^k^TKA: total knee arthroplasty.

^l^UKA: unilateral knee arthroplasty.

^m^Currency conversions calculated on May 24, 2022.

Chen et al [58] developed a blended intervention comprising 4 in-person group sessions of health education and exercise and telephone follow-up, with the remaining sessions at home. Although the blended intervention of Cuperus et al [73] comprised 2 in-person group exercise sessions and 4 telephone calls from a specialized nurse, the blended intervention in Kloek et al [59] comprised 5 in-person PT and home exercises using a website that provided education along with a graded activity and exercise. All 3 studies found similar improvements in either self-reported symptoms or physical activity between those who received blended interventions and those who received health education [58] or in-person PT [59,73]. In another RCT, Kloek et al [59] reported statistically significant and clinically nonsignificant improvements at 3 and 12 months in physical function and physical activity with a 3-month blended intervention (in-person PT sessions+website with incremental physical activity program, exercise, and education) compared with in-person usual PT. De Vries et al [62] then used data from the blended intervention arm of this RCT to investigate factors related to the adherence to the digital component of the blended intervention. The authors observed the highest adherence for participants with middle (vs low or high) education level, duration of symptoms of 1 to 5 years (vs <1 year or >5 years), and those recruited by physical therapists [62]. Other factors positively related to adherence included participants’ internet skills, self-discipline, the execution of the exercise plan and usability, flexibility, design, added value, and time required for the digital intervention [62]. Thus, although blended interventions may elicit improvements similar to in-person PT, a number of individual and program-related factors are associated with adherence to the web-based component of blended interventions.

Baker et al [60] developed a 2-year telephone-based intervention comprising the assessment of exercise behavior, goal setting, counseling, and alerts when exercise adherence lapsed but found similar improvements in exercise adherence in those who received the telephone intervention and those who received automated reminder messages to exercise. Similarly, Hinman et al [61] found similar improvements in overall knee pain in those who received telephone counseling from both nurses and PT and in those who received counseling from nurses only. Despite the nonsignificant improvements in pain, Hinman et al [61] found statistically significant but clinically nonsignificant improvements in function, satisfaction, and adherence to the telephone intervention. Lawford et al [71] speculated that these clinical improvements in participants might be associated with their relationship with PT. However, secondary analysis of the data revealed only weak associations between therapeutic alliance and improvements in pain, function, and fear of movement [71].

#### Directly Supervised Exercise Interventions for Populations With KR: Real-time Videoconferencing, Multi-Technology, and Telephone-Based

In individuals before and after KR, digital health PT interventions were mostly investigated as replacements for traditional in-person PT (Table 4).

Doiron-Cadrin et al [63] found high satisfaction and clinically meaningful within-group improvements in pain and function with a 12-week prehabilitation program using real-time videoconferencing, which were similar to those in people who received the 12-week prehabilitation program in person. Similar outcomes between digital and in-person PT interventions have also been reported in individuals after KR for video-based and inertial motion sensor–based digital health interventions [63-66]. However, some outcomes (physical activity, muscle strength, exercise behavior, climbing stairs, walking, and body pain) favored in-person PT at longer follow-up periods (2, 4, 12, or 18 months after the intervention) [65,52]. Moreover, better outcomes with digital health than with in-person PT have been seen when using multi-technology platforms, with improvements in pain and function [67-69] above the MCID [55,75] and persisting even at longer follow-up periods of 3 and 6 months [68,69]. This suggests that these intensive multi-technology digital interventions may be more effective than simpler digital health interventions. These multi-technology platforms included motion-tracking sensors paired with a mobile app for biofeedback; a website portal to report activity to a therapist who could modify the exercise program as needed; or motion-tracking cameras with an avatar for exercise delivery, outcome reporting, and clinician monitoring [67-69]. Finally, a retrospective study found a high response rate (92%), satisfaction (89%), and acceptability (87%) for an internet-based rehabilitation follow-up [74]. However, the lack of comparison with in-person follow-up limits the conclusions on the efficacy of internet-based follow-ups in this population.

### Digital Health for Psychological Interventions for Chronic Pain Management

#### Overview

In addition to patient education and exercise, there is growing evidence showing the efficacy of behavioral interventions such as CBT and PCST for the management of chronic pain because of KOA [7,9]. This section includes digital interventions that incorporated such psychological treatments (Table 5). A detailed description of the studies and the technology used in the studies is shown in Table S5 in Multimedia Appendix 1 [76-87].

**Table 5:** Digital health for psychological interventions.

Study	Population	Design	Intervention		Comparator or comparators		Primary outcome findings
			Description	Sample size	Description	Sample size	
Nevedal et al [80]	Chronic pain, including osteoarthritis	Pre or post design	Commercially available web-based program	645	None	N/A^a^	Improvements in pain intensity and pain unpleasantness on a 0- to 10-point Likert scale
Rini et al [79]	Hip or knee osteoarthritis	RCT^b^	PainCoach (internet-based PCST^c^)	58	No intervention	55	Significant improvements in pain on the Arthritis Impact Measurement Scale 2
Bennell et al [76]	Chronic knee pain	RCT	Website for education and PCST program and videoconferencing for exercises delivered by PT^d^	74	Website for education	74	No difference in improvements between groups for the NRS^e^ pain score and the WOMAC^f^ function at 6 months
Lawford et al [85]	Chronic knee pain	Exploratory analyses from an RCT	Website for education and PCST program and videoconferencing for exercises delivered by PT	74	Website for education	74	Greater improvements for the NRS pain score in employed people in the intervention vs employed people in the comparator; greater NRS pain improvements in people who had higher self-efficacy
Mecklenburg et al [77]	Chronic knee pain	RCT	Inertial movement sensors and tablet computer with an app that includes an exercise plan, CBT^g^, weight loss, personal coach, and peer support	101	Digitally delivered patient education	61	Greater improvements for the KOOS^h^ pain and function in the intervention vs comparator
O’Moore et al [78]	Knee osteoarthritis with major depressive disorder	RCT	Internet-based CBT program)+usual treatment	44	Usual treatment	25	Improvements in intervention for depression and psychological distress
Stome et al [81]	Osteoarthritis	Pre or post	12-week goal achievement program using behavior change app Vett (personalized goals+2-3 corresponding weekly tasks decided during an in-person consultation with physician+self-monitoring+cues and reminders+individual feedback and communication with an assigned mentor)	12	N/A	N/A	High levels of acceptability, utility, and usability
Bennell et al [82]	Knee osteoarthritis and obesity	2-group superiority RCT (TARGET trial)	24-week behavior change, theory-informed, automated, SMS text messaging interventions that address barriers to and facilitators of adherence	56	No SMS text messaging	54	Greater improvements in exercise adherence on the Exercise Adherence Rating Scale in the intervention vs comparator
Dharmasri et al [83]	African Americans with osteoarthritis	Mixed methods RCT: data from the intervention arm of the trial	STAART^i^ trial: 11-session, telephone-based PCST program delivered by counselors+ handouts+audio recording for progressive muscle relaxation	93	N/A	N/A	Participants found the program helpful and described the following themes: improved pain coping, mood and emotional benefits, improved physical functioning, and experiences related to intervention delivery
Pronk et al [87]	Post-KR^j^	Unblinded RCT	PainCoach app that gave advice on pain medication use, exercise or rest, and when to call the clinic in response to a patient’s input of pain experienced	38	Same advice as PainCoach given in usual care	33	No difference between groups in improvements in pain at rest, during activity, or at night
Buvanendran et al [86]	Pre-KR	RCT	8-week telehealth CBT and 4-week telehealth CBT	30 (8 weeks) and 20 (4 weeks)	4-week in-person CBT and no CBT	15 (4 week) and 15 (no CBT)	Improvements in PCST but not WOMAC pain scores in the intervention vs comparator
McCurry et al [84]	Moderate to severe osteoarthritis and insomnia	RCT	Telephone-based 8-week CBT for insomnia+daily sleep diaries+sleep hygiene education+cognitive strategies	136	Education related to living with chronic osteoarthritis	146	Improvement on Insomnia Severity Index in the intervention vs comparator

^a^N/A: not applicable.

^b^RCT: randomized controlled trial.

^c^PCST: pain coping skills training.

^d^PT: physical therapy.

^e^NRS: Numeric Pain Rating Scale.

^f^WOMAC: Western Ontario and McMaster Universities Osteoarthritis Index.

^g^CBT: cognitive behavioral therapy.

^h^KOOS: Knee Osteoarthritis Outcome Score.

^i^STAART: Skills Training for African Americans with Osteoarthritis study

^j^KR: knee replacement.

#### Digital Health for Psychological Interventions for Populations With KOA: Websites, Mobile App, Text Messaging, Multi-Technology, Telephone-Based, and Real-time Videoconferencing

These technologies (website: 3/91, 3%; telephone: 1/91, 1%; SMS text messages: 1/91, 1%; mobile apps 1/91, 1%; real-time videoconferencing: 1/91, 1%) typically included features such as easy-to-use interfaces, tailored goal-setting and daily assignments, education, behavioral coaching by animated characters or by counselors, reminders, activity and sleep logs, wearable sensors for tracking movement, and communication with clinicians. Although the content of these interventions varied, overall, all studies that included CBT or PCST showed statistically and clinically meaningful small to medium improvements in knee pain, as reported by MCID and effect sizes, with a digital health intervention [76-84]. Furthermore, in people with KOA who also met the criteria for major depressive disorder, web-based CBT (6 web-based lessons, regular homework assignments, access to supplementary sources, and contact with clinical psychologists if scores on self-reported outcome measures deteriorated significantly) along with usual treatment was found to be more effective than usual treatment alone in improving depression symptoms and psychological health, in addition to improving pain, function, and self-efficacy [78]. Similarly, in people with KOA who also had insomnia, an 8-week telephone-based CBT intervention comprising six 20- to 30-minute telephone calls, sleep hygiene education, and techniques to reduce hyperarousal and nonsleep activities in bed at night improved insomnia, pain, and fatigue immediately after treatment, which were sustained at the 12-month follow-up [84]. However, these improvements in pain did not reach clinical significance [84]. Despite these promising results, none of these studies compared digital interventions alone with in-person interventions; hence, it is not clear whether digital interventions for chronic pain management are noninferior or superior to in-person interventions in people with KOA. In addition, in an exploratory study, employment and self-efficacy—but not age, education, expectation of outcome, BMI, or pain catastrophizing—appeared to moderate the effects of a 3-month digital health program on pain [85], suggesting that these factors may be considered when assessing the effectiveness of these interventions.

#### Digital Health for Psychological Interventions for Populations With KR: Mobile App, Telephone, and Real-time Videoconferencing

Psychological treatment such as a 4-week telehealth CBT for people with high pain catastrophizing scores undergoing KR showed moderate improvements in psychological health (pain catastrophizing scores), which did not translate to clinical improvements in pain [86]. In contrast, an unblinded RCT investigated a digital health intervention *PainCoach*, which coached or provided advice to the patient on what to do in response to the patient’s input of pain and showed a statistically significant reduction in opiate use but nonsignificant improvements in pain compared with usual care [87]. However, given the preliminary nature of these studies and the limited number of studies in KR populations, definitive conclusions regarding the efficacy of psychological interventions delivered by digital health in KR populations cannot be made.

### Cost-effectiveness of Digital Health

Another important component in understanding the utility of digital health in KOA or KR is the relative costs of these programs. A detailed description of the studies included in this section is provided in Table S6 of Multimedia Appendix 1 [74,88-93].

#### Cost-effectiveness of Digital Health Interventions for People With KOA

We identified 2% (2/91) of studies that explicitly focused on cost-effectiveness analyses of digital health interventions for KOA (Table 6). These studies used data from clinical trials described previously in this review. Kloek et al [92] reported that a 12-week blended intervention for patients with hip osteoarthritis or KOA comprising 5 in-person PT sessions and a website program with education, exercise, and a graded activity module had lower intervention and medication costs but similar societal and health care costs than in-person PT. It should be noted that similar improvements were seen in both groups, despite the participants in the digital arm receiving 7 fewer sessions on average than those in the in-person arm [92]. In contrast, Cuperus et al [88] reported that a multidisciplinary in-person intervention to improve self-management skills was slightly more cost-effective than a blended intervention of 2 PT group sessions and 4 telephone calls (€387 [US $483.62] vs €252 [US $314.92], respectively) in patients with generalized osteoarthritis. Given the differences in study design (eg, populations, components of digital interventions, and comparators) and the overall lack of research in this area, it is challenging to draw any conclusions regarding the cost-effectiveness of digital health interventions for people with KOA.

**Table 6:** Cost-effectiveness of digital health.

Study	Population	Design	Intervention		Comparator or comparators		Findings
			Description	Sample size	Description	Sample size	
Cuperus et al [88]	Generalized osteoarthritis	RCT^a^	2 in-person group sessions+telephone monitoring by nurse	72	Multidisciplinary in-person group intervention led by PT^b^	75	No difference in quality-adjusted life years and total societal costs
Kloek et al [92]	Knee or hip osteoarthritis	RCT	Website+in-person PT	108	Usual in-person PT	99	Lower intervention costs and medication costs for intervention vs comparator but no difference in total societal and health care costs
Marsh et al [89,90]	Post-KR^c^ or HR^d^	RCT	Web-based platform to schedule patient visits	118	Usual protocol to schedule visits	111	Lower costs for intervention vs comparator
Tousignant et al [91]	Post-KR	RCT	Custom hardware with videoconferencing and remote-controlled cameras	97	In-person home-based PT	100	Lower costs for intervention vs comparator
Fusco et al [93]	Post-KR	Markov decision modeling	10 videoconferencing sessions and 10 in-person PT sessions	—^e^	20 in-person PT sessions	—	High probability of the intervention group being cost-effective, particularly when transportation was included
El Ashmawy et al [74]	Post-KR or HR	Retrospective study	Remote joint replacement clinic follow-up at 1-year, 7-years, and every 3-years after in-person consultations at 2 weeks and 6-weeks	1749	N/A^f^	N/A	Estimated saving of £42,644 (US $53,439.93) per year with intervention

^a^RCT: randomized controlled trial.

^b^PT: physical therapy.

^c^KR: knee replacement.

^d^HR: hip replacement.

^e^Not available.

^f^N/A: not applicable.

#### Cost-effectiveness of Digital Health Interventions for People With KR

For people after KR, 5% (5/91) of studies suggested that digital health reduces patient and societal costs [74,89-91,93,94]. Marsh et al [89] evaluated the costs of a web-based follow-up, comprising web-based questionnaires following x-rays, email reminders, and alerts to schedule in-person appointments if necessary, and reported that after 1 year from surgery, digital health was more cost-effective than in-person follow-up after KR because of reduced travel and associated costs [90] and from a societal and health care perspective. Similarly, El Ashmawy et al [74] reported that remote follow-ups at longer postoperative periods (after a 1-year postoperative period) were more cost-effective than in-person follow-ups. Furthermore, 2% (2/91) of studies compared videoconferencing with or without in-person PT with in-person PT and reported that telerehabilitation was cost-effective when transportation costs were included in the analysis [91,93]. In individuals before KR, a mobile app–based prehabilitation intervention that provided individualized exercises, progressions, and daily pain monitoring was more cost-effective than no prehabilitation as the prehabilitation program reduced the length of hospital stay (7.6 vs 11.9 days) and consequently reduced hospital costs [94]. However, in this case, the reduced costs could be attributed to prehabilitation and not necessarily to digital health.

### Patient and Clinician Perspectives on Digital Health

#### Patients’ Perspectives on Digital Health

To determine the potential of digital health for KOA, an understanding of the patient and clinician perspectives on these technologies is needed. Several studies have reported patient and clinician perspectives on a variety of digital health interventions (Table 7).

Overall, patients with KOA had positive experiences with digital health technologies. Some of the key benefits noted by patients included anonymity, accessibility, convenience, tailored interventions, reduced travel costs, feedback and self-monitoring, progress reports, and enhanced patient-provider relationships [95-105]. Phone-based interventions were found to be acceptable and were valued for the undivided focus and communication from physical therapists [96-98]. However, some patients who lacked confidence in their exercise technique wanted some form of visual supervision (videoconferencing) to be incorporated into their exercise intervention [97]. Although people with KOA had positive views about digital health technologies, they also discussed some concerns related to navigating these technologies. These concerns typically included challenges with the user interface, dislike for repetitive reminders and texts, lack of variation in exercises, accommodation for comorbidities (eg, decreased motor coordination and visual and hearing impairments), privacy and security, preference for customized notification, need for technological support, willingness to pay, and lack of in-person contact with clinicians [81,96,98-104,106]. Despite this, people with KOA were willing to use a digital program whether it was endorsed by their health care professional or by a credible organization [99-102].

**Table 7:** Patient and clinician perspectives on digital health.

Technology	Patient perspectives	Clinician perspectives
Telephone interventions [96,97,107,108]	Willing to use Less acceptable than videoconferencing	More acceptable after first-hand experience Liked the focus on communication and self-management rather than manual therapy Less acceptable than videoconferencing Lack of visual cues and difficulty with examination Requires training
Telerehabilitation and real-time videoconferencing [65,98]	Acceptable, feasible, and satisfactory Improved access and relationship with the therapist Preferred over telephone Convenience, ease of use, and privacy More patient-focused than in-person visits No consensus about willingness to pay Requires technological assistance	High satisfaction with goal achievement, patient-therapist relationships, and quality and performance Liked that patients may be more active in managing their disease Preferred over telephone Discomfort with lack of physical contact Lack of experience can lead to low confidence and reduced interest
Websites [90,95,99-101]	Moderate to high satisfaction Cost and time savings Anonymity, accessibility, and flexibility Similarly preferred as in-person for scheduling visits Preferred over social media, group self-management programs, or telephone helplines Increased acceptance if endorsed by a health care professional Monitoring progress, access to information, feedback from health care professionals, and connecting with peers May depend on technological capabilities Real-life avatar preferred over animation Nonnative accents not preferred; desire for more context and culture specific	Professional autonomy and added value to practice Effective, acceptable, and feasible Apprehensive of extra time needed to incorporate digital health, especially during high workload Need for flexibility to tailor to an individual Need for training Financial concerns
Mobile app [102,103]	Prefer big buttons, tapping vs sliding, and vertical vs horizontal layout Progress feedback reports and educational tips High levels of acceptability, user satisfaction, and technical usability Useful for self-management and improved communication with physicians Do not prefer extra clicking, complicated user interface, and unnecessary information	Liked the weekly or monthly pain and activity reports Prioritized precision of presentation and interpretation of questions Useful for patient resources and accountability Skepticism because of the need for internet access at the clinic and technological aptitude
Smartwatch app [104]	Interest in direct phone call capability, weather apps, and health-tracking sensors such as accelerometer and heart rate sensor Concerns regarding usability, accessibility, notification customization, and intuitive user design	—^a^
Social media [109]	Limited prior experience among participants Less preferred compared with web-based and mailed information packs	—
Wearable biofeedback system [110]	—	Useful for movement feedback, monitoring, and adherence Challenges with monitoring, reliability, information accuracy, and individualization

^a^Not available. No relevant studies were identified.

#### Clinician’s Perspectives on Digital Health

Clinicians also noted the benefits of digital health technologies but appeared more likely than patients to identify challenges. Although accessibility and convenience were noted as positive aspects, there were concerns related to implementation, apprehension about the technology, lack of physical contact, data protection, lack of digital health and communication training, and revenue loss [98,102,107,108,110,111]. Hurley et al [112] showed that appropriate training can lead to improvements in physical therapists’ knowledge, skills, confidence, and the delivery of digital health interventions. Similar to patients, clinicians preferred video-based over telephone-based interventions [107]. However, training and experience were found to improve clinicians’ perspectives on telephone-based interventions [108]. Physical therapists also found value in monitoring patients’ data, particularly in being able to track movements, but were concerned with adoption in patients who may not be technologically proficient [103,110]. Furthermore, health care professionals discussed wanting more information on patients’ compliance to exercise, relevant outcomes, and validity of tracking with the digital health program [113]. Interestingly, one of the studies noted that physicians did not support the use of mobile apps as they considered KOA to be a minor problem, were concerned about their involvement, and needed the internet at the clinic [102]. These findings provide opportunities for further improvements in digital health interventions based on patients’ and clinicians’ perspectives.

## Discussion

### Principal Findings

Digital health has been used to provide patient education, physical activity, and exercise interventions (self-directed, remotely monitored, or directly supervised by a clinician), as well as psychological interventions such as CBT and PCST, in people with KOA and KR. The types of digital health used for these purposes included websites, telephone calls, SMS text messaging, mobile apps (with or without visible feedback from activity monitors), real-time videoconferencing, and multi-technology systems that combined a few different technologies in their intervention. These technologies were typically used in place of or to augment in-person clinical care. Multiple technologies were often combined (eg, activity monitoring with mobile apps and wearable sensors with websites) in digital interventions to leverage the strengths of multiple technologies. Overall, we found substantial heterogeneity in the types of digital health interventions that have been investigated for people with KOA and KR.

Only a few recent studies on the use of digital health for patient education were identified [23,24,26-28,35]. Although these studies found improvements in disease-related knowledge—with digital interventions providing patient education—in people with KOA and KR [23,24,28], the clinical meaningfulness of these improvements is unclear. Irrespective of the technology used for the dissemination of patient education, all studies in KR populations found improvements in disease- and surgery-related knowledge in users before their KR or soon after their KR [26,27,35]. However, the studies in KR populations were limited (3/91, 3%), and it is also not clear whether these results hold true at longer follow-up periods (ie, a few months after KR surgeries). In both the KOA and KR populations, it was noted that providing regular and person-specific information (eg, via push notifications in a mobile app or SMS text messaging) in contrast to general advice and relying on patients to access the information at their convenience may lead to improved disease-related knowledge [23,27,35]. It was also identified that publicly available content on social media may have incomplete or misleading information that could further erode patient trust in the information provided via digital means [29-32,34].

In people with KOA, the benefits of digital health for exercise and physical activity interventions in people with KOA appear mixed. In contrast, in people with KR, many studies reported significant improvements in self-reported outcomes with digital exercise interventions that were similar to in-person treatments [63-66]. Although the different technologies used in these studies (eg, websites, telephone, mobile apps, videoconferencing, and multi-technology systems) were generally acceptable to people with KOA and KR, some participants who used telephone-based interventions stated the need for visual contact with their physical therapists [96-98]. However, currently, research comparing different modes of intervention delivery using different technologies is lacking. Overall, it appears that interventions that use >1 technology and strategies to engage the participants (eg, activity monitoring with a mobile app, activity monitoring with motivational messaging, and telephone coaching) may be more promising than those that rely on a single modality (eg, website or SMS text messaging) [39,46,47,67]. For interventions delivered by physical therapists to people with KOA, blended interventions that use digital health strategies to augment in-person care may provide benefits similar to those of in-person care [41,59,70,73]. However, more research that directly compares blended, digital, and in-person care is required to comprehensively understand the potential of blended interventions. Digital health interventions that include CBT or PCST components have shown statistically significant and clinically meaningful improvements in outcomes in patients with KOA and KR. However, there is a lack of research comparing these approaches with traditional in-person approaches; thus, conclusions cannot be drawn about how they compare with in-person psychological interventions for chronic pain management. Finally, although digital health appears to be cost-effective when compared with in-person treatments, research on the cost-effectiveness of digital health is too limited to draw definitive conclusions.

### Comparison With Prior Work

Choi et al [18] conducted a systematic review of mobile apps for osteoarthritis self-management. The authors concluded that digital health tools for the self-management of osteoarthritis mostly provided patient education and lacked rigorous evidence. They recommended that future mobile apps should include self-management, decision support, and shared decision-making as key functionalities for people with osteoarthritis. Our review expands on this prior work as we included all available types of digital health (eg, social media and websites) versus only mobile apps. Our findings show that these tools improve patient knowledge; however, whether they translate into improved outcomes is not clear. Safari et al [21] also published a systematic review and meta-analysis of digital self-management interventions for people with KOA. They included interventions delivered via telephone plus audio and video, the internet, or mobile apps. They concluded that moderate-quality evidence suggests small to medium improvements in pain and function immediately after the intervention, which was sustained at 12 months. They included studies of self-guided exercise interventions as part of their analyses and considered any comparator (eg, usual care, other digital health, alternative treatment, and no treatment). Although we did not undertake a meta-analysis, our review provides more nuances and context by teasing out the findings by type of intervention (eg, education and self-guided exercise) and comparator (eg, in-person exercise).

In people with KR, 2 prior systematic reviews reported greater improvements in pain, function, knee extension, and quadricep strength in people who received digital interventions than in those who received in-person PT [17,19], whereas another reported similar improvement in knee range of motion, physical activity, and function in people who received post-KR rehabilitation in person or by telerehabilitation [16]. Our review extends these results by including studies that investigated a range of digital health technologies (websites, mobile apps, SMS text messages, phone based, and synchronous and asynchronous videoconferencing). Furthermore, the findings of our review build on existing literature by noting that digital interventions for people with KR, which incorporated multi-technology platforms, were associated with statistically and clinically significant improvements in pain and function [55,74,75], which persisted even at longer follow-up periods of 3 and 6 months [74,75]. Hence, our review extends the findings reported in some prior studies and captures important advances in digital health spurred by the onset of the COVID-19 pandemic, when remote health care greatly expanded [2].

### Limitations

There are a few limitations to be considered when interpreting the findings of this review. First, a comparison of specific digital health technologies (eg, websites vs mobile apps) or their components was beyond the scope of this review. Second, the focus of our review was on studies that used digital health for interventions in KOA and KR and thus did not address other applications of digital health such as informed consent, movement assessment, diagnosis, and data collection. Third, this review focused only on the primary outcomes reported in the included studies. Additional insights may be gained by reviewing the secondary and exploratory outcomes. Fourth, as this was not a systematic review, these findings should be interpreted with caution. The intent of this literature review is to provide researchers and clinicians with an overview of the digital health interventions currently used for KOA and KR. Finally, as our last search was conducted in February 2021; studies published after this date were not included in this review.

### Future Directions

This review shows that digital health has promising potential in the future of health care for people with KOA and KR. For readability and quality of digitally delivered education, it may be valuable for digital interventions to curate content from credible websites, treatment guidelines, or cocreate educational resources with people with KOA. Moreover, the information provided by digital interventions should be validated by licensed health care providers before it is disseminated to patients. For physical activity and exercise interventions, future studies should consider leveraging existing knowledge of patient and clinician preferences while developing and implementing digital health approaches. Furthermore, given that user engagement and adherence remain a challenge in this population, providing technological support (eg, phone calls and easy-to-use user interface) and clinical support (eg, communication with a clinician via asynchronous or synchronous chats, phone, or video calls) could improve the adoption of digital health technologies in people with KOA. In addition to providing technological and clinical support, other patient-related contextual factors such as employment, educational attainment, and eHealth literacy, should be considered while prescribing digital treatments to ensure greater adherence [62,85]. Specific technological preferences in terms of intervention flexibility and user experience in the reviewed studies may also be important when prescribing digital health interventions [81,101,104,106,109,114]. Flexibility in interventions that allow for some degree of personalization, such as activating or deactivating features based on personal preferences and the ability to alter intervention design based on comorbidities (eg, visual impairments and hand osteoarthritis), may also foster adherence [106]. From the clinician’s perspective, reimbursement models that incentivize the use of digital health interventions are needed [115]. Although these findings provide some guidance, the best practice would be to include all stakeholders (clinicians and patients) while developing new digital health interventions [116]. For example, researchers or research organizations could liaise with patient organizations to understand preferred sources of information (eg, YouTube videos) and lead efforts to improve the quality and readability of information available through those sources. Finally, concerns regarding privacy and data security continue to be raised by both patients and clinicians. Therefore, transparent disclosure of how data generated from digital health platforms will be used and kept secure may be vital for their uptake in real-world settings.

### Conclusions

In conclusion, digital health offers exciting opportunities for improving care delivery for people with KOA or KR. For people with KOA and KR, interventions that are blended (digital health and in person), incorporate multiple technologies, patient monitoring or visible biofeedback, and communication with clinicians may have more favorable outcomes. However, comparative studies investigating the different technologies are lacking. Future implementation of these promising technologies should consider incorporating patient and clinician preferences into the digital health intervention design process.

## Appendix

Multimedia Appendix 1 Detailed description of studies included in the review.

## Abbreviations

CBT: cognitive behavioral therapy
IBET: Internet-Based Exercise Therapy
KOA: knee osteoarthritis
KR: knee replacement
MCID: minimal clinically important difference
PATH-IN: Physical Therapy versus Internet-Based Exercise Training
PCST: pain coping skills training
PT: physical therapy
RCT: randomized controlled trial
WOMAC: Western Ontario and McMaster Universities Osteoarthritis Index
