# Supplementary material for: Applications of Digital Health Technologies in Knee Osteoarthritis: Narrative Review
Source: JMIR Rehabil Assist Technol. 2022 Jun 8;9(2):e33489. doi: 10.2196/33489 (PMC9218886; doi:10.2196/33489)
Supplement: Multimedia Appendix 1 [file rehab_v9i2e33489_app1.docx]

**APPENDIX: TABLES**

**Table S1: Digital health for patient education in knee osteoarthritis**

| **Brosseau et al, 2014[28]** | |
| --- | --- |
| Study Design | - Design = Pre/post - Purpose = Improvement in knowledge, skills, and self-efficacy through educational material on Facebook - Population = OA or RA; participated in intervention (n) = 99; lost to follow-up (n) = 14 |
| Intervention | - Modality = “Group” web pages on Facebook - Features = videos uploaded from YouTube on how to apply self-management interventions, URL for online questionnaires, and regular reminders to fill questionnaires - Duration = 2 weeks |
| Primary Outcome | - Customized online surveys on “Survey Monkey” = Knowledge of self-management interventions, intent and actual use of self-management strategies, self-efficacy - Time period of assessment = Immediately post-intervention (2 weeks) |
| Findings | - Knowledge acquisition scores (mean difference in improvement)   - In-depth topics = 0.8 (*P*≤.05)   - Introductory topics = 1 (*P*≤.01)   - Combined topics = 1.8 (*P*<.01) - Self-management strategies (%)   - Intent = 83%   - Actual use = 100% for ice massage and aquatic therapy - Self-efficacy (mean (SD))   - Post-intervention scores out of 10 = 4.55 (3.39) - 7.65 (2.40) |
| Limitations | - Self-reported OA/RA - No comparator group |
| **Umapathy et al, 2015[24]** | |
| Study Design | - Design = Quasi-experimental design - Purpose = evaluate quality of care and self-management after access to a publicly available website “My Joint pain” - Population = Hip/knee OA; participated in intervention (n) = 277; lost to follow-up (n) = 82 |
| Intervention | - Modality = website - Features = credible and tailored information regarding OA, monthly assessments with validated instruments, progress tracking, use as per participant’s convenience - Duration = 12 months - Comparator = participants who did not use the website (n=91) |
| Primary Outcome | - heiQ - Time period of assessment = post-intervention (12 months) |
| Findings | - Between-group difference - No significant difference between groups in all domains of heiQ - Within-group difference - All domains except emotional distress, constructive attitudes, and health service navigation showed significant improvements |
| Limitations | - Large attrition - Data related to disease severity and technology usage was not collected - Sampling bias |
| **Timmers et al, 2018[23]** | |
| Study Design | - Design = RCT - Purpose = improvement in disease-related knowledge after patient education on a mobile/tablet app - Population = Knee pain; participated in intervention (n) = 91; lost to follow-up (n) = 14 |
| Intervention | - Modality = “Patient Journey” app accessible on mobile phone or tablet - Features = daily push notifications using text, photos, and videos, interactive quizzes assessing disease-related knowledge - Duration = 7 days before consultation with orthopedic surgeon - Comparator = Information offered during medical consultations (n = 122) |
| Primary Outcome | - Custom questionnaires = level of perceived and actual knowledge about knee osteoarthritis and treatment options - Time period of assessment = 2 days before the consultation |
| Findings | - Between group difference, mean (SD) - Perceived Knowledge = 26% higher in app group (app: 16.5 (3.9), control: 13 (4.1), *P*<.001) - Actual Knowledge = 52% higher in app group (app: 26.4 (7.4), control: 17.4 (6.8), *P*<.001) |
| Limitations | - 22% of patients in the intervention group did not download the app - Use of self-created questionnaires |
| **Wang et al, 2020[25]** | |
| Study Design | - Design = Quasi-experimental study - Purpose = evaluate quality of care and health education after access to the updated “My Joint Pain” website - Population = Hip/ Knee OA; participated in intervention (n) = 35 |
| Intervention | - Modality = “My Joint Pain” updated website - Features = credible and tailored information regarding OA including library with videos and fact sheets, monthly assessments with validated instruments, progress tracking, use as per participant’s convenience - Duration = 12 months - Comparator = Participants who did not use the website (n=87) |
| Primary Outcome | - heiQ, OAQI - Time period of assessment = 12 months from when the website was updated |
| Findings | - Between group changes - heiQ = No significant change in either domains between users and non-users - OAQI = better quality of care in treatment alternatives (12%), self-management (17%), NSAIDs (13%), and referral to orthopedic surgeons (23%) in users compared to non-users of the website |
| Limitations | - Large attrition – only 44% of participants who had used the original website responded to the use of the updated version of “My Joint Pain” - Lack of randomization - Did not maintain grouping for the whole study |
| **Fraval et al, 2015[26]** | |
| Study Design | - Design = RCT - Purpose = evaluate whether access to education website along with standard consent process improves quality of informed consent - Population = Pre-surgery (KR or HR); participated in intervention (n) = 103 |
| Intervention | - Modality = access to education website along with standard discussion with surgeon - Features = overview of surgical procedure, diagnosis and indications of surgery, complications, and pre-, intra-, and post-operative care - Comparator = Standard discussion with orthopedic surgeon related to consent (n = 108) |
| Primary Outcome | - Custom questionnaire = knowledge about operation |
| Findings | - Difference between groups, %(SD) - Correct answers knowledge related to operation = intervention: 69.25% (14.91), control: 47.38% (17.77), *P*<.01 |
| Limitations | - Lack of longitudinal follow-up |
| **Campbell et al, 2019[27]** | |
| Study Design | - Design = RCT - Purpose = evaluate effectiveness of a SMS bot with respect to patient engagement - Population = Post-surgery (KR or HR); participated in intervention (n) = 76; lost to follow-up (n) = 0 |
| Intervention | - Modality = SMS on phone - Features = automated texts with recovery instructions and encouraging messages, personalized video messages from surgeon, and short instructional therapy videos along with traditional education - Duration = 6 weeks - Comparator = pre-operative clinical appointment and perioperative instructions (n=83) |
| Primary Outcome | - Time spent participating in home exercises - Time period of assessment = 6 weeks from KR surgery |
| Findings | - Mean difference between groups, mean (95% CI) - Time exercising per day (min) = 8.6 (4.9, 12.4) *P*<.001 |
| Limitations | - Response bias owing to the self-reported data - Selection bias as participants from only a single orthopedic center were included |
| **Timmers et al, 2019[35]** | |
| Study Design | - Design = RCT - Purpose = evaluate effect of an interactive app on pain compared to traditional patient education - Population = Post-surgery (KR)  ; participated in intervention (n) = 114 ; lost to follow-up (n) = 7 |
| Intervention | - Modality = Patient Journey App - Features = daily personalized education tailored to the patient’s phase of recovery - Duration = daily for 4 weeks - Comparator = basic information about recovery process twice a week (n=99) |
| Primary Outcome | - NRS pain scores at rest, during activity, and at night - Time period of assessment = weekly in first 4 weeks after discharge |
| Findings | - Difference between groups, intervention vs control, mean at 4 weeks   - Pain at rest = 3.45 vs 4.59, *P* =.001   - Pain during activity = 3.99 vs 5.08, *P*<.001   - Pain at night = 4.18 vs 5.21, *P*=.003 |
| Limitations | - Only 82% downloaded the app - Risk of bias due to custom questions from hospital staff instead of direct feedback from patients |
| **Meldrum et al. 2017[29]** | |
| Study Design | - Purpose = cross-sectional quality content analysis - Population = knee pain |
| Intervention | 1000 random comments from 58 YouTube videos related to non-specific knee pain |
| Primary Outcome | Categorizing nature of comments |
| Findings | Comments with personal information (19%), appreciation or acknowledgement (17%), and asking questions or seeking help (15%) |
| Limitations | Findings of the study restricted to people who actively seek information online |
| **Barrow et al. 2018[30]** | |
| Study Design | - Design = cross-sectional quality content analysis - Purpose = assess quality of OA education content on websites |
| Intervention | Websites with OA-specific content (n=50) |
| Primary Outcome | Osteoarthritis Quality Proforma |
| Findings | 68% of the websites scored more than half of the maximum available quality score; median total score = 41 |
| Limitations | - Non-standardized and non-validated assessment tool - Limited websites as search was done only in UK-based search engines |
| **Murray et al. 2019[32]** | |
| Study Design | - Design = cross-sectional quality content analysis - Purpose = assess readability and quality of OA education content on websites |
| Intervention | Websites on OA-specific content (n=37) |
| Primary Outcome | - Readability on Flesch Reading Ease Score, Flesch-Kincaid Grade Level, and Gunning-Fog Index - Quality on Journal of the American Medical Association benchmark criteria and DISCERN criteria |
| Findings | - Readability varied from 8^th^ to 12^th^ grade level - 2.7% of websites met all 4 Journal of American Medical Association criteria - Mean DISCERN quality score = “fair” |
| Limitations | - Only analyzed websites from the top three search engines - Some degree of subjectivity with quality assessment |
| **Chapman et al. 2019 [31]** | |
| Study Design | - Design = cross-sectional quality content analysis - Purpose = assess accessibility in terms of readability of information provided on health websites for knee OA |
| Intervention | Health education websites for knee OA (n=49) |
| Primary Outcome | Standardized readability indexes = SMOG, FOG |
| Findings | - Of 49, only 5 websites met the recommended readability levels for health education literature - 44.9% of web pages did not include informative images to support text - Only 6.12% included relevant videos |
| Limitations | - Readability assessment lacks measure of comprehension - Accessibility and quality indicators such as format, font, and user-interface of websites were not considered |
| **Wong et al, 2019[34]** | |
| Study Design | - Design = cross-sectional quality content analysis - Purpose = assess educational quality of YouTube videos on knee OA and KR |
| Intervention | YouTube videos on knee OA (n=56) and KR (n=50) |
| Primary Outcome | Custom educational quality assessment checklist on a scale of 0-10  Poor Quality = 0-3, Acceptable quality = 4-7, Excellent quality = 8-10 |
| Findings | - Knee OA videos = 66% poor quality, 32% acceptable quality, 2% good quality - KR videos = 64% poor quality, 28% acceptable quality, 8% good quality |
| Limitations | - Lack of validated standardized instruments - No grading for inaccurate information in videos |
| **Bahadori et al, 2020 [33]** | |
| Study Design | - Design = cross-sectional quality content analysis - Purpose = assess readability of information provided within HR and KR apps |
| Intervention | Mobile apps targeting total hip and/or knee replacement surgeries (n=15) |
| Primary Outcome | Standardized readability indexes = GFI, GRES, FKGL |
| Findings | - Readability scores, mean (SD) - GFI = 9.1 (1.4) - FRES = 56.9 (11.3) - FKGL = 9.7 (1.3) - Of 15, only 1 app (My THR) was “easy-to-read” |
| Limitations | - Selection bias because only free apps in English language were included - Only a small body of text was analyzed from the main page in the app |
| FOG=Gunning Fog Index; FKGL=Flesch-Kincaid Grade Level; FRES=Flesch Reading Ease Score; heiQ=Health Education Impact Questionnaire; HR=Hip Replacement; KR=Knee Replacement; NRS=Numeric Rating Scale; OA=Osteoarthritis; OAQI=Osteoarthritis Quality Indicator; RA=Rheumatoid arthritis; RCT=Randomized Controlled Trial; SD=standard deviation; SMOG=Simple Measure of Gobbledegook; SMS=Short Messaging Services; THR = Total Hip Replacement; URL=Uniform Resource Locator | |

**Table S2: Digital Health for Physical Activity Interventions in People with Knee Osteoarthritis**

| **Bossen et al, 2013[38]** | |
| --- | --- |
| Study Design | - Design = pre/post single-arm pilot study - Purpose = determine preliminary effectiveness of “Join2move” website intervention - Population = knee/hip OA; participated in intervention (n) = 20; lost to follow-up (n) = 9 |
| Intervention | - Modality = website - Features = fully automated text-messaging and emails, password-protected, free, available 24/7, self-paced, specific short-term PA goals based on baseline test of recreational activity selected by participant, 8 tailored weekly modules with increasing difficulty level, flexibility in terms of repeating the module or changing difficulty levels. - Duration = 9 weeks |
| Primary Outcome | - Physical activity (SQUASH), self-perceived effect and physical function (KOOS) - Time period of assessment = 6- and 12-weeks |
| Findings | - Post-intervention 12-weeks vs Baseline scores, mean (SD) - Physical activity = 2044 (1369) vs 1697 (1174) - Pain = 5.2 (1.8) vs 5.3 (1.7) - Function, ADL = 47.5 (20.6) vs 46.8 (20.1) |
| Limitations | - Small sample size - Lack of long-term follow-up - Self-reported PA data may have resulted in overestimation of PA levels - People with low computer or internet access skills were excluded |
| **Li et al, 2017[41]** | |
| Study Design | - Design = RCT - Purpose = evaluate feasibility and preliminary efficacy of wearable activity monitor and PA counseling by PT - Population = knee OA; participated in intervention (n) = 17; lost to follow-up (n) = 0 |
| Intervention | - Modality = Telephone calls, wearable activity monitor - Features = group education session on benefits of physical activity and detrimental effects of sedentary behavior, weekly PA telephone counseling by PT, wearable activity monitor (Fitbit Flex) - Duration = 4 weeks - Comparator = Same intervention delayed by 1 month (n=17) |
| Primary Outcome | - Feasibility = rates of recruitment, drop-outs, and adherence - Preliminary efficacy = minutes of MVPA per day - Time period of assessment = post-intervention (Month 1, Month 2) |
| Findings | - Feasibility - Rates of recruitment = 74%, drop-outs = 0, adherence = all but 1 participant - Minutes of MVPA per day at month 1, mean (SD) - Intervention group = 64.2 (70.5); Control group = 56 (60.1) - Minutes of MVPA per day at month 2, mean (SD) - Intervention group = 67.7 (85.8); Control group = 81.9 (64.4) |
| Limitations | - Limited generalizability as participants were relatively active at baseline |
| **Skrepnik et al, 2017[36]** | |
| Study Design | - Design = RCT - Purpose = evaluate impact of mobile app plus activity monitor on mobility - Population = people with knee OA treated with hylan G-F 20; participated in intervention (n) = 107; lost to follow-up (n) = 3 |
| Intervention | - Modality = mobile app (OA GO) plus wearable activity monitor - Features = provided motivational messages, collected pain, mood, and physical activity data once daily - Duration = 90 days - Comparator = usual care plus wearable activity monitor with no visible feedback (n=104) |
| Primary Outcome | - Change in steps per day - Time period of assessment = post-intervention (day 90) |
| Findings | - Change in steps per day, intervention vs control - Mean number change = 1199 vs 467, *P*=0.03 - Mean percentage change = 35.8% vs 11.5%, *P*=0.02 |
| Limitations | - Selection bias as people with low and high levels of physical activity at baseline were excluded - No long-term follow-up |
| **Bartholdy et al, 2019[37]** | |
| Study Design | - Design = RCT - Purpose = evaluate effect of motivational text messages on sedentary behavior - Population = knee OA; participated in intervention (n) = 19; lost to follow-up (n) = 1 |
| Intervention | - Modality = Text-messages - Features = 3 text-messages per week on importance of daily physical activity - Duration = 6-weeks - Comparator = No attention (n=19) |
| Primary Outcome | - Time spent physically inactive measured by tri-axial accelerometer - Time period of assessment = post-intervention (6-weeks) |
| Findings | - Mean difference in change between groups, mean (95% CI) - Time spent inactive (min/day) = 13.2 (-41, 67.3) |
| Limitations | - Small sample size in individual groups |
| **Zaslavsky et al, 2019[39]** | |
| Study Design | - Design = pre/post pilot study - Purpose = evaluate feasibility and preliminary efficacy of a mhealth intervention - Population = people with OA and disturbed sleep; participated in intervention (n) = 24; lost to follow-up (n) = 2 |
| Intervention | - Modality = phone calls, text-messages, activity monitor - Features = wearable activity monitor (Fitbit charge 2), 3 phone calls for motivational interviewing, weekly personalized text-messages for motivational feedback based on their step count attainment, self-monitoring through Fitbit cloud server - Duration = 14 weeks |
| Primary Outcome | - Feasibility = number of participants eligible, enrolled, retained - Preliminary efficacy = ISI; secondary measure = total step count measured - Time period of assessment = baseline, 14 weeks, 19 weeks |
| Findings | - Feasibility - Number of participants eligible=24, enrolled=24, retained=22 - Longitudinal change at 19 weeks, estimate (95% CI) - ISI = -1.24 (-2.43, -0.05) - Total step count (step/days) = 44 (-321, 410) |
| Limitations | - Selection bias as participants who were ‘very active’ or who did not own a smartphone were excluded - No control group - No objective outcome measures |
| **Allen et al, 2020[40]** | |
| Study Design | - Design = pre/post pilot study - Purpose = evaluate feasibility, acceptability, and preliminary efficacy of OA-PCP - Population = knee/hip OA; participated in intervention (n) = 67; lost to follow-up (n) = 14 |
| Intervention | - Modality = phone calls and emails - Features = PA screening, 3 PA coaching phone calls including goal-setting based on preferences and availability of resources, check-in emails with linkage to community or online resources, phone-call follow-up - Duration = 3 months |
| Primary Outcome | - Feasibility = number of participants eligible, enrolled, and retained - Acceptability = Survey question on 0-10 scale - Preliminary efficacy = minutes of MVPA measured by accelerometer - Time period of assessment = 4 months |
| Findings | - Feasibility - Number of participants eligible = 70, enrolled = 67, retained = 53 - Acceptability, mean SD - Perceived helpfulness of OA-PCP program = 7.64 (2.5) - Mean difference at 4 months (95% CI) - Minutes of MVPA = 0.78 (-1.6, 3.2) |
| Limitations | - Low external validity as participants from only one health system were enrolled limiting generalizability - Not sufficiently powered to find significant differences in outcome measures - No control group |
| CI = Confidence Interval; ISI = Insomnia Severity Index; MVPA = Moderate-Vigorous Physical Activity; OA = osteoarthritis; OA-PCP = Osteoarthritis Physical Activity Care Pathway; PA = Physical Activity; PT = Physical Therapist; RCT = Randomized Controlled Trial; SD = Standard Deviation; SQUASH = Short Questionnaire to Assess Health-enhancing physical activity | |

**Table S3: Self-directed or Asynchronous Digital Exercise Interventions**

| **Dahlberg et al, 2016[44]** | |
| --- | --- |
| Study Design | - Design = pre/post pilot study - Purpose = evaluate effect of a web-based self-management program “Joint Academy” - Population = knee/hip OA; participated in intervention (n) = 53; lost to follow-up (n) = 17 |
| Intervention | - Modality = website - Features = video-based lectures on education related to OA, physical activity, and self-management strategies, post-lecture quizzes, weekly tailored 12-14 neuromuscular exercises, and pain reports - Duration = 6 weeks |
| Primary Outcome | - Knee pain = 0-10 NRS scale - Time period of assessment = Week 6, 12, 18, 24, and 30 |
| Findings | - Post-intervention scores, mean (SD); baseline = 5.1 (2.1) - Week 6 = 4.5 (1.8); Week 12 = 3.6 (2.0); Week 18 = 3.3 (2.5); Week 24 = 2.7 (1.7); Week 30 = 3.2 (2.1) |
| Limitations | - High drop-out rate - No control group |
| **Nero et al, 2017[45]** | |
| Study Design | - Design = observational and quasi-experimental study - Purpose = evaluate clinical outcomes of a web-based self-management program “Joint Academy” - Population = knee/hip OA; participated in intervention (n) = 350; lost to follow-up (n) = 100 |
| Intervention | - Modality = website - Features = “Joint Academy” program – education, tailored exercises, email reminders, 2 telephone consultations with physical therapist. - Duration = 6 weeks - Comparator = Results from annual reports of Swedish in-person self-management program “Better Management of Patients with Osteoarthritis (BOA)” |
| Primary Outcome | - Knee pain = 0-10 NRS score - Time period of assessment = 6-week (within group), 3 months (between group) |
| Findings | - Within-group change in NRS pain score at 6-weeks, mean (SD) - Baseline = 5.4 (2.2); Post-intervention = 4.1 (2.4) - Pain scores in intervention and control group at 3 months, mean (SD) - Joint Academy = 3.5 (2.2); BOA = 37 on a 0-100 VAS scale |
| Limitations | - Lack of randomization – no direct comparison |
| **Allen et al, 2018[52]** | |
| Study Design | - Design = RCT - Purpose = evaluate effectiveness of in-person PT and IBET compared to wait-list control - Population = knee OA; participated in intervention (n) = 142; lost to follow-up (n) = 30 |
| Intervention | - Modality = Website - Features = tailored exercises with progressions based on individual pain and function, automated reminders, progress tracking including graphs of pain, function, and exercise - Duration = 12 months - Comparator = in-person PT (n= 140); wait-list control (n=68) |
| Primary Outcome | - WOMAC total score - Time period of assessment = 4-months, 12-months |
| Findings | - Difference in baseline to 4-months vs wait-list, (95% CI) - IBET = -2.70 (-6.24, 0.85) - In-person PT = -3.36 (-6.84, 0.12) - Difference in baseline to 12-months vs wait-list, (95% CI) - IBET = -2.63 (-6.37, 1.11) - In-person PT = -1.59 (-5.26, 2.08) |
| Limitations | - Physical therapists were allowed to vary interventions between participants - Low external validity as sample did not include participants with low education level |
| **Pignato et al, 2018[43]** | |
| Study Design | - Design = secondary analysis from RCT (Allen et al, 2018) - Purpose = evaluate associations between treatment dose and outcomes in IBET and in-person PT - Population = knee OA; intervention group included in analyses (n) = 135 |
| Intervention | - Modality = Website - Features = tailored exercises with progressions based on individual pain and function, automated reminders, progress tracking including graphs of pain, function, and exercise - Duration = 4 months - Comparator = in-person PT (n= 124) |
| Primary Outcome | - Association between number of PT visits and amount of IBET use with WOMAC total score - Time period of assessment = 4 months |
| Findings | - Model without interaction, improvement in WOMAC total score, estimate (95% CI) - Number of PT visits = -1.18 (-1.91, -0.46) - Number of days on IBET website = -0.06 (-0.14, 0.01) |
| Limitations | - No measurement of exercise participation in the IBET group |
| **Nelligan et al, 2021[42]** | |
| Study Design | - Design = RCT - Purpose = evaluate efficacy of a web-based strengthening exercise program “My Knee Exercise” - Population = knee OA; participated in intervention (n) = 103; lost to follow-up (n) = 13 |
| Intervention | - Modality = website - Features = website access to education regarding knee OA, importance of exercise and physical activity, guidance to improve physical activity, self-directed knee strengthening program, automated text-messages providing adherence support - Duration = 24 weeks - Comparator = website access to only educational content (n=103) |
| Primary Outcome | - Knee pain = 0-10 NRS scale - Physical function = WOMAC function subscale (0-68) - Time period of assessment = 24 weeks |
| Findings | - Mean difference in change between groups, (95% CI) - Knee pain = 1.6 (0.9, 2.2) - Physical function = 5.2 (1.9, 8.5) |
| Limitations | - Selection bias as all participants included had favorable views on technology, high levels of education, and high self-efficacy at baseline - Not clear if participants in control group participated in exercise or physical activity |
| **Dahlberg et al, 2020[6]** | |
| Study Design | - Design = longitudinal cohort study - Purpose = evaluate long-term clinical outcomes of a web-based self-management program “Joint Academy” - Population = knee/hip OA from the registry of the program; included in analysis (n) = 499 at 24-weeks, (n) = 138 at 48-weeks |
| Intervention | - Modality = website - Features = video-based lectures on education related to OA, physical activity, and self-management strategies, post-lecture quizzes, weekly tailored 12-14 neuromuscular exercises, and pain reports - Duration = 48 weeks |
| Primary Outcome | - Pain = 0-10 NRS scale - Function = 30CST - Time period of assessment = 24-weeks, 48-weeks |
| Findings | - 24-week sub-sample, mean scores (95% CI) - Pain at baseline = 5.6 (5.4, 5.8); 24-weeks = 3.1 (2.9, 3.4) - Function at baseline = 10 (9.6, 10.4); 24-weeks = 14.3 (13.7, 14.8) - 48-week sub-sample, mean scores (95% CI) - Pain at baseline = 5.7 (5.2, 6.1); 24-weeks = 2.9 (2.4, 3.5); 48-weeks = 3.2 (2.7, 3.8) - Function at baseline = 10.3 (9.6, 10.9); 24-weeks = 15.1 (13.9, 16.3); 48-weeks = 14.4 (13.1, 15.7) |
| Limitations | - Lack of control group for direct comparison |
| **Gohir et al, 2021[53]** | |
| Study Design | - Design = compare efficacy of “Joint Academy” with usual self-management care - Purpose = RCT - Population = knee OA; participated in intervention (n) = 63; lost to follow-up (n) = 15 |
| Intervention | - Modality = website - Features = tailored neuromuscular exercises, education related to OA and self-management, quizzes, adherence by emails, smartphone notifications, or via telephone or asynchronous chat with physical therapist - Duration = 6 weeks - Comparator = usual care given by general practitioner or physical therapist (n= 57) |
| Primary Outcome | - Knee pain = 0-10 NRS scale - Time period of assessment = 6 weeks |
| Findings | - Between group difference in change, mean (95% CI) - Pain = -1.5 (-2.2, 0.8) |
| Limitations | - Exercises or physical activity performed by the control group were not monitored |
| **Piqueras et al, 2013[51]** | |
| Study Design | - Design = RCT - Purpose = compare effectiveness of interactive telerehabilitation system with in-person PT - Population = post-KR; participated in intervention (n) = 90; lost to follow-up (n) = 22 |
| Intervention | - Modality = interactive virtual software-hardware platform - Features = interactive software with 3D avatar demonstrating exercises, motion sensors to track exercises, web portal for therapist to monitor data, telephone calls from therapist if necessary - Duration = 2 weeks - Comparator = in-person PT (n=91) |
| Primary Outcome | - Active ROM - Time period of assessment = 2 weeks, 3 months |
| Findings | - Difference from baseline at 2 weeks, intervention vs control, mean (SD) - Active knee flexion = 8.53 (6.56) vs 7.71 (6.89), *P*=0.29 - Active knee extension = 0.2 (2.8) vs 0.9 (3.7), *P*=0.045 - Difference from baseline at 3 months - Active knee flexion = 18.16 (9.71) vs 15.63 (8.82), *P*=0.19 - Active knee extension = 1.3 (3.1) vs 0.8 (3.3), *P*=0.47 |
| Limitations | - Short duration of intervention - No active tracking of physical activity other than exercises |
| **Bini et al, 2017[50]** | |
| Study Design | - Design = RCT - Purpose = compare effectiveness of an asynchronous video platform with in-person PT - Population = post-KR; participated in intervention (n) = 13 |
| Intervention | - Modality = mobile app - Features = app allowed video uploads by physical therapist (instructions) and participants (recording of themselves completing the exercises) - Duration = no prescribed end point - Comparator = in-person PT (n=15) |
| Primary Outcome | - PROMs = KOOS, VAS, VR-12 - Time period of assessment = 3 months following surgery |
| Findings | - Change from pre-op to post-op scores, mean (SD) - KOOS = -17.4 (15.4) - VAS = -3.7 (2.9) - VR-12 physical component = 15.3 (10.1) - VR-12 mental component = 3.6 (10.6) |
| Limitations | - Small sample size - Potential difference in exercise participation because there was no structured endpoint of intervention - Significant differences in follow-up time frame between patients |
| **Chughtai et al, 2019[46]** | |
| Study Design | - Design = pre/post study - Purpose = evaluate efficacy of a prehabilitation program prior to TKA - Population = pre-KR; participated in intervention (n) = 114 |
| Intervention | - Modality = mobile app “PreHab” - Features = checklists with tasks to complete before the surgery, education on pain management, daily exercise, nutrition advice, mindfulness program, interaction with peers - Comparator = participant who did not use the mobile app (n= 362) |
| Primary Outcome | - Hospital length of stay, discharge disposition (home without assistance, home with health aide, or nursing facility) - Time period of assessment = post-surgery length of hospital stay |
| Findings | - Hospital length of stay, mean (range) - Prehabilitation group = 2 (1-5) - control group = 2.7 (1-9) - Discharge disposition, prehabilitation vs controls - Home without assistance = 77.2% vs 42.8%, *P*<0.001 - Home with health aide = 21.1% vs 31.8%, *P*=0.04 - Nursing facility = 1.8% vs 21.8%, *P*<0.0001 |
| Limitations | - Analysis did not control for baseline characteristics, comorbidities, or knee OA severity |
| **Fleischman et al, 2019[49]** | |
| Study Design | - Design = randomized non-inferiority trial - Purpose = evaluate effectiveness of unsupervised home exercise after TKA - Population = post-KR; participated in intervention (n) = 96; lost to follow-up (n) = 16 |
| Intervention | - Modality = website - Features = weekly exercises with online video demonstrations, interactive patient monitoring and communication portal - Duration = 8 weeks - Comparator = in-person PT (n=97); printed exercise manual (n) = 91 |
| Primary Outcome | - Change in passive knee flexion measured by goniometer - Time period of assessment = from baseline to 4-6 weeks |
| Findings | - Adjusted difference in change in passive knee flexion based on pre-defined 5° margin of in-person PT (95% CI) - Web-based PT = +3° (-1.2°, 6.4°) - Paper PT = + 5° (0.99°, -8.6°) |
| Limitations | - Potential selection bias as participants may have included mostly motivated people |
| **Klement et al, 2019[48]** | |
| Study Design | - Design = Retrospective chart review at a single institution - Purpose = evaluate efficacy of self-directed PT after TKA - Population = post-KR; participated in intervention (n) = 296; successful completion of program (n) = 195 |
| Intervention | - Modality = website - Features = web-based platform sends email with weekly exercises including written descriptions, pictures, and videos of the exercises - Duration = 10 weeks - Comparator = failure of completion of program (i.e.) <90° knee flexion at 2-weeks post-op with self-reported function limitations (n=101) |
| Primary Outcome | - Completion of self-directed exercise program - Time period of assessment = post-intervention, 10 weeks |
| Findings | - 65.9% did not require in-person PT post-intervention - 34.1% were prescribed in-person PT following the self-directed program due to limited knee range of motion (66.3%), patient request (27.7%), and inability to use website platform (1%) |
| Limitations | - Lack of education in the intervention might account for the high request (27.7%) of in-person PT - Lack of standardized questionnaires |
| **Ramkumar et al, 2019[47]** | |
| Study Design | - Design = pre/post pilot study - Purpose = evaluate feasibility of remote patient monitoring mobile app - Population = pre-KR; participated in intervention (n) = 25; lost to follow-up (n) = 3 |
| Intervention | - Modality = mobile app “TKR” paired with motion sensors in wearable knee sleeve - Features = reminders to complete exercises, movement tracking (knee sleeve), avatar-based biofeedback (ROM data from motion sensors paired with Bluetooth on app), pain reports - Duration = 3 months |
| Primary Outcome | - Mobility = step count - ROM = knee flexion - PROMs = KOOS - Time period of assessment = 3 months post-op |
| Findings | - Steps per day, mean (range) = 4654 (1154-12108) - Knee flexion, mean = 119° - KOOS improvement, mean (range) = 39.3 (3-60) |
| Limitations | - Selection bias as all participants in the study had access to and were proficient with smartphones - Small sample size |
| 30CST = 30-second chair stand test; CI = Confidence Interval; IBET = Internet Based Exercise Therapy; KR = Knee replacement surgery; KOOS = Knee Osteoarthritis Outcome Score; NRS = Numeric Rating Scale; OA = Osteoarthritis; PT = Physical Therapy; PROM = Patient Reported Outcome Measure; RCT = Randomized Controlled Trial; ROM = Range Of Motion; SD = Standard Deviation; TKA = Total Knee Arthroplasty; VAS = Visual Analog Scale; VR-12 = Veterans Rand 12-item health survey; WOMAC = Western Ontario and McMaster Universities Osteoarthritis Index Score | |

**Table S4: Digital health for Directly Supervised Exercise Interventions**

| **Cuperus et al, 2015[73]** | |
| --- | --- |
| Study Design | - Design = RCT - Purpose = compare effectiveness of telephone based and in-person self-management program - Population = generalized OA; participated in intervention (n) = 77; lost to follow-up (n) = 5 |
| Intervention | - Modality = blended (telephone + in-person group sessions) - Features = 2 in-person group education sessions, 4 telephone contacts with rheumatology nurse for goal setting, activity pacing, and pain management advice - Duration = 6 weeks - Comparator = in-person treatment (n=81) |
| Primary Outcome | - Daily function = HAQ-DI - Time period of assessment = week 6, 26, and 52 |
| Findings | - Mean difference in HAQ-DI change scores between groups - ∆ 6 weeks = 0.01 (-0.10. 0.13) - ∆ average 1 year = -0.03 (-0.14, 0.07) |
| Limitations | - No measure to ensure that participants performed the exercises - Lack of no-attention control group |
| **Bennell et al, 2017[70]** | |
| Study Design | - Design = RCT - Purpose = evaluate efficacy of additional telephone coaching to home-based PA program - Population = knee OA; participated in intervention (n) = 84; lost to follow-up (n) = 14 |
| Intervention | - Modality = blended (telephone + home PT sessions) - Features = 6 behavioral change coaching calls, 5 sessions of home-based exercise delivered by PT - Duration = 6 months - Comparator = 5 sessions of home-based exercise delivered by PT (n=84) |
| Primary Outcome | - Knee pain = NRS scale (0-10) - Function = WOMAC function subscale (0-68) - Time period of assessment = 6 months |
| Findings | - Post-intervention scores, intervention vs control, mean (SD) - Pain = 3.1 (2.2) vs 3.8 (2.3) - Function = 14.7 (10.6) vs 18.2 (11.7) |
| Limitations | - Large attrition rate |
| **Kloek et al, 2018[59]** | |
| Study Design | - Design = RCT - Purpose = compare short- and long-term effectiveness of e-exercise and in-person PT - Population = knee/hip OA; participated in intervention (n) = 109; lost to follow-up (n) = 20 |
| Intervention | - Modality = blended (online application + in-person individual sessions) - Features = 5 in-person session with physical therapist, e-exercise included graded activity goals, strength and stability exercises, weekly education videos, automatic reminder emails - Duration = 12 weeks - Comparator = in-person PT (n=99) |
| Primary Outcome | - Physical function = KOOS function, TUG test - Time period of assessment = 3 months, 12 months |
| Findings | - Between group mean difference (95% CI) - KOOS function 3 months = -1.4 (-5.6, 2.8); 12 months = -0.2 (-6.4, 6.0) - TUG 3 months = 0.3 (-0.8, 1.5) |
| Limitations | - Selection bias as all participants were high functioning at baseline - High dropout rates and missing data |
| **De Vries et al, 2018[62]** | |
| Study Design | - Design = mixed-method study embedded within RCT (Kloek et al.) - Purpose = determine factors related to adherence in online component of blended treatment program - Population = knee/hip OA; included in analyses (n) = 90 |
| Intervention | - Modality = online application - Features = graded activity goals, strength and stability exercises, weekly education videos, automatic reminder emails - Duration = 12 weeks |
| Primary Outcome | - Quantitative = Regression analysis to determine factors related to adherence - Qualitative = Individual semi-structured interviews (n=10) - Time period of assessment = 12 weeks |
| Findings | - Regression analysis, β-coefficient, p value - Middle education = -0.397, p=0.264 - OA duration 1-5 years = -0.873, *P*=0.02 - Recruited by physical therapist = 0.627, *P*= 0.03 - Qualitative analysis - Sufficient internet skills, self-discipline, execution of exercise plan, intervention’s usability, flexibility, design, time required, and research participation were linked with adherence |
| Limitations | - Not clear if participants who used the online application actually performed the exercises |
| **Chen et al, 2019[58]** | |
| Study Design | - Design = quasi-experimental trial - Purpose = compare effectiveness of a home-based exercise program to education only - Population = knee OA; participated in intervention (n) = 84; lost to follow-up (n) = 13 |
| Intervention | - Modality = blended (4 in-person group sessions + home exercises) - Features = group education and exercise sessions, home exercises, exercise diary and telephone calls from research assistants to encourage adherence - Duration = 12 weeks - Comparator = education sessions and handouts, telephone follow-ups (n=87) |
| Primary Outcome | - WOMAC pain and stiffness - Time period of assessment = post-intervention (12 weeks) |
| Findings | - Post-intervention scores, intervention vs control - WOMAC pain, mean (SD) = 4.28 (3.30) vs 5.73 (3.54) - WOMAC stiffness, median (interquartile range) = 1 (0,3) vs 2 (1,4) |
| Limitations | - Lack of randomization - Selection bias as sample did not include participants with severe baseline pain (NRS>7) - Lack of long-term follow-up |
| **Baker et al, 2020[60]** | |
| Study Design | - Design = RCT - Purpose = evaluate effect of BOOST-TLC program on adherence - Population = knee OA; participated in intervention (n) = 52; lost to follow-up (n) = 7 |
| Intervention | - Modality = telephone - Features = assessment of exercise behavior, exercise goal setting, counseling messages, alerts to research team and information on lapsing if participant was not exercising, motivational calls, monthly automated reminder messages on phone to exercise - Duration = 2 years - Comparator = monthly automated reminder messages on phone to exercise (n=52) |
| Primary Outcome | - Self-reported adherence on a 0-10 scale - Time period of assessment = post-intervention (2 years) |
| Findings | - Post-intervention scores, intervention vs control - Adherence, mean (range) = 3.63 (2.70, 4.56) vs 4.01 (3.03. 4.99) - Difference in change, mean (95% CI) = -0.38 (-1.67, 0.91) |
| Limitations | - Self-reported adherence |
| **Doiron-Cadrin et al, 2020[63]** | |
| Study Design | - Design = pilot RCT - Purpose = evaluate feasibility of a tele-prehabilitation program - Population = pre-KR/HR; participated in intervention (n) = 12; lost to follow-up (n) = 1 |
| Intervention | - Modality = real-time videoconferencing - Features = supervised exercise sessions with physical therapist twice per week - Duration = 12 weeks - Comparator = in-person prehabilitation (n=11); usual care (n=11) |
| Primary Outcome | - Feasibility = recruitment rate, number of telerehabilitation sessions performed, technical issues, compliance to prehabilitation programs, satisfaction - Time period of assessment = post-intervention (12 weeks) |
| Findings | - Recruitment rate = Of 111 screened over 6 months, 34 participants were recruited - Number of telerehabilitation sessions = 191 (Reacts Lite app, Skype, and Facetime) - Technical issues = inability to log in, sporadic sound, image freezing, internet related connectivity issues - Compliance = 77% for tele-prehabilitation group, 80% in-person group - Satisfaction = 91% generally comfortable, 91% found tele-prehabilitation as good as in-person care |
| Limitations | - Small sample size - Selection bias as participants needed to have access to high-speed internet in order to participate in study |
| **Hinman et al, 2020[61]** | |
| Study Design | - Design = RCT - Purpose = evaluate efficacy of additional telephone-based exercise advice and support intervention to existing nurse consultation - Population = knee OA; participated in intervention (n) = 87; lost to follow-up (n) = 5 |
| Intervention | - Modality = telephone - Features = 5-10 consultations with physical therapist over phone, information folder, exercise bands, access to a website for exercise videos - Duration = 6 months - Comparator = ≥1 nurse consultation for self-management advice (n=88) |
| Primary Outcome | - Knee pain on 0-10 NRS scale - WOMAC physical function (0-68 scale) - Time period of assessment = post-intervention (6 months) |
| Findings | - Post-intervention scores, intervention vs control, mean (SD) - Knee pain = 3.5 (2.1) vs 4.2 (2.2) - WOMAC function = 18.4 (11.3) vs 22 (12.5) |
| Limitations | - Unclear if participants performed any physical activity outside of the intervention - No activity/exercise tracking during the intervention to ensure participants performed their exercises |
| **Lawford et al, 2021[71]** | |
| Study Design | - Design = secondary analysis from RCT (Hinman et al.) - Purpose = evaluate association between therapeutic alliance and clinical outcomes following a telephone delivered intervention - Population = knee OA; included in analysis (n) = 87 |
| Intervention | - Modality = telephone - Features = 5-10 consultations with physical therapist over phone, information folder, exercise bands, access to a website for exercise videos - Duration = 6 months |
| Primary Outcome | - Therapeutic Alliance = WAI - Clinical outcomes = knee pain (NRS), function (WOMAC), self-efficacy (ASES), quality of life (AQoL), adherence (11-point scale) - Time period of assessment = 6 and 12 months |
| Findings | - Patient rating of therapeutic alliance, regression co-efficient (95% CI) - Knee pain 6 months = -0.10 (0.16, 0.03); 12 months = -0.06 (-0.13, 0) - Function 6 months = -0.10 (-0.40, 0.20); 12 months = -0.13 (-0.43, 0.18) - Self-efficacy 6 months = 0.16 (0.04, 0.28); 12 months = 0.15 (0.03, 0.27) - Quality of life 6 months = 0.01 (0.01, -0.01); 12 months = 0.01 (-0.01, 0.01) - Adherence to exercise 6 months = 0.09 (-0.02, 0.20); 12 months = 0.04 (-0.07, 0.15) |
| Limitations | - All outcome measures were self-reported - WAI has not been validated for use in PT |
| **Russell et al, 2011[72]** | |
| Study Design | - Design = RCT - Purpose = compare efficacy of a telerehabilitation program with in-person PT - Population = post-KR; participated in intervention (n) = 31; lost to follow-up (n) = 1 |
| Intervention | - Modality = real-time videoconferencing - Features = 45-min exercise and education session delivered by physical therapists - Duration = 6 weeks - Comparator = in-person PT (n=34) |
| Primary Outcome | - WOMAC - Time period of assessment = post-intervention (6 weeks) |
| Findings | - Difference between groups at 6-weeks (95% CI) - Pain = 0.78 (-0.26, 1.83) - Stiffness = 1.46 (0.24, 2.68) - Function = 1.07 (-0.01, 2.14) - Total = 1.10 (0.14, 2.07) |
| Limitations | - Lack of long-term follow up - Limited external validity as telerehabilitation software including internet was controlled by the research team |
| **Tousignant et al, 2011[65]** | |
| Study Design | - Design = RCT - Purpose = compare effectiveness of telerehabilitation and in-person rehabilitation - Population = post-KR; participated in intervention (n) = 24; lost to follow-up (n) = 3 |
| Intervention | - Modality = real-time videoconferencing - Features = 16 exercise videoconferencing sessions delivered by physical therapist - Duration = 2 months - Comparator = in-person PT (n=24) |
| Primary Outcome | - Disability = knee extension ROM - Function = WOMAC function subscale - Time period of assessment = post-intervention (2 months) |
| Findings | - Mean difference between groups - Knee extension: 1.1°, *P*>0.05 - WOMAC: 8.1 points, *P*>0.05 |
| Limitations | - Need for high-speed internet and remote controlled cameras might not be feasible in real-life setting |
| **Moffet et al. 2015[64]** | |
| Study Design | - Design = RCT - Purpose = compare efficacy of telerehabilitation program with home PT visits - Population = post-KR; participated in intervention (n) = 104; lost to follow-up (n) = 10 |
| Intervention | - Modality = real-time videoconferencing - Features = 16 sessions of exercise and education sessions delivered by physical therapists - Duration = 4 months after KR hospital discharge - Comparator = home PT visits (n=101) |
| Primary Outcome | - WOMAC - Time period of assessment = 4 months after hospital discharge |
| Findings | - Difference between groups at 4 months (95% CI) - Pain = -1.6 (-5.9, 2.8) - Stiffness = -0.7 (-6.8, 5.4) - Function = -1.8 (-5.9, 2.3) - Total = -1.6 (-5.6, 2.3) |
| Limitations | - Limited external validity as telerehabilitation software including internet was controlled by the research team |
| **Correia et al, 2018[69]** | |
| Study Design | - Design = feasibility study - Purpose = compare efficacy of digital biofeedback system with in-person home-based rehabilitation - Population = post-KR; participated in intervention (n) = 38; lost to follow-up (n) =8 |
| Intervention | - Modality = motion trackers, mobile app, website - Features = inertial motion trackers to track exercise movements and provide real-time feedback regarding exercise on mobile app, remote clinical monitoring through web-based platform - Duration = 8 weeks - Comparator = in-person home-based rehabilitation (n=31) |
| Primary Outcome | - TUG - Time period of assessment = post-intervention (8-weeks) |
| Findings | - Change within group, intervention vs control - TUG median (IQR) = -9.5 (8.0) vs -4.6 (8.6), p=0.04 |
| Limitations | - Small sample size - Lack of true randomization – participants were allocated using a geographical criteria |
| **Correia et al, 2019[68]** | |
| Study Design | - Design = feasibility study - Purpose = evaluate medium-term outcomes of a digital biofeedback system - Population = post-KR; participated in intervention (n) = 38; lost to follow-up (n) =8 |
| Intervention | - Modality = motion trackers, mobile app - Features = inertial motion trackers to track exercise movements and provide real-time feedback regarding exercise on mobile app, remote clinical monitoring through web-based platform - Duration = 8 weeks - Comparator = in-person home-based rehabilitation (n=31) |
| Primary Outcome | - TUG - Time period of assessment = 3- and 6-months post KR |
| Findings | - Change from baseline (95% CI) - TUG 3 months = -4.48 (-1.64, -7.37); 6 months = -4.87 (-1.85, -7.47) |
| Limitations | - Small sample size - Lack of true randomization – participants were allocated using a geographical criteria |
| **Bell et al, 2020[66]** | |
| Study Design | - Design = feasibility study - Purpose = evaluate feasibility of additional remote rehabilitation monitoring platform (interACTION) to in-person PT - Population = post-KR; participated in intervention (n) = 13; lost to follow-up (n) = 3 |
| Intervention | - Modality = motion trackers, mobile app - Features = inertial motion trackers, color-coded biofeedback on mobile app, written and video instructions for exercise, web-based clinical portal, adherence tracking - Duration = 10 weeks - Comparator = in-person PT + unsupervised home exercise program (n=12) |
| Primary Outcome | - Value = change in ADLS of Knee Outcome Survey/ total cost of rehabilitation - Time period of assessment = post-intervention (10 weeks) |
| Findings | - Post-intervention, intervention vs control - Value = 0.013 ± 0.007 (8) vs 0.018 ± 0.009 (8), *P*=0.32 |
| Limitations | - Small sample size |
| **Chughtai et al, 2020[67]** | |
| Study Design | - Design = pre/post feasibility study - Purpose = evaluate efficacy of a Virtual Exercise Rehabilitation Assistant (VERA) - Population = post-KR; participated in intervention (n) = 157 |
| Intervention | - Modality = telerehabilitation system - Features = clinician prescribed PT protocols through animated display, 3-D motion tracking cameras, real-time feedback, remote monitoring through clinician portal - Duration = no prescribed end point |
| Primary Outcome | Total time spent exercising |
| Findings | Total time spent exercising, mean = 29.5 days; 26.5 min per day |
| Limitations | - Small sample size - Short follow-up period |
| **El Ashmawy et al, 2021[74]** | |
| Study Design | - Design = Retrospective study - Purpose = evaluate effectiveness of virtual follow-up - Population = post-KR/HR; included in analyses (n) = 1749 |
| Intervention | - Modality = VJRC package, telephone calls - Features = VJRC package (instruction letter, PROM booklet, information regarding local radiology for X-Rays), telephone review of PROM and X-Ray outcomes - Duration = in-person consultations at 2-weeks and 6-weeks; virtual follow-up at 1-year, 7-year, and every 3 years |
| Primary Outcome | Patient Response rate, satisfaction, outcome, costs |
| Findings | - Patient Response rate = 92.05% overall response rate - Satisfaction = 89.29% were satisfied - Outcome = 7.22% required in-person appointment with 3% reviewed by orthopedic consultant - Costs = estimated saving of £42, 644 annually |
| Limitations | - Retrospective cohort and direct comparison with in-person PT not possible |
| ADLS = Activities of Daily Living Scale; AM-PAC = Boston University Activity Measure for Post-Acute Care; ASES = Arthritis Self-Efficacy Scale; AQoL = Assessment of Quality of Life; BOOST-TLC = Boston Overcoming Osteoarthritis through Strength Training Telephone-Linked Communication; CI = Confidence Interval; HAQDI = Health Assessment Questionnaire Disability Index; HR = Hip Replacement; IQR = Inter-Quartile Range; KOOS = Knee Osteoarthritis Outcome Score; KR = Knee Replacement; KSS = Knee Society Score; NRS = Numeric Rating Scale; PROM = Patient Reported Outcome Measures; PT = Physical Therapy; RCT = Randomized Controlled Trial; ROM = Range of Motion; SUS = System Usability Score; OA = Osteoarthritis; TUG = Timed Up and Go; VJRC = Virtual Joint Replacement Clinic; WAI = Working Alliance Inventory-Short Form; WOMAC = Western Ontario and McMaster Universities Osteoarthritis Index Score | |

**Table S5: Digital health for Psychological Interventions**

| **Nevedal et al, 2013[80]** | |
| --- | --- |
| Study Design | - Design = pre/post design - Purpose = evaluate effectiveness of a web-based chronic pain management program - Population = chronic pain including OA; participated in intervention (n) = 645 |
| Intervention | - Modality = web-based program - Features = commercially available content through text, images, videos and interactive tools: education, tailored action plan and access to pain management tools based on baseline questionnaire, ongoing feedback, reminders - Duration = no prescribed end point |
| Primary Outcome | - Knee pain intensity and unpleasantness = 0-10 NRS scale - Time period of assessment = 1- and 6-months from baseline |
| Findings | - Difference from baseline, Cohen’s d - Pain intensity 1 month = -0.21, *P*=0.008; 6 months = -0.22, *P*=0.005 - Pain unpleasantness 1 month = -0.25, *P*=0.002; 6 months = -0.25, *P*=0.002 |
| Limitations | - Unclear if participants exercised as per the self-selected program as exercise compliance was not measured - Possible regression to mean as the study was uncontrolled - Lack of control group |
| **Rini et al, 2015[79]** | |
| Study Design | - Design = pilot RCT - Purpose = evaluate efficacy of internet based PCST (PainCOACH) - Population = knee/hip OA; participated in intervention (n) = 58; lost to follow-up (n) = 5 |
| Intervention | - Modality = web-based program - Features = virtual coach teaching behavioral coping skills through weekly interactive modules, reminder and encouragement messages, badges for completion of modules, self-monitoring - Duration = 8 weeks - Comparator = No attention control (n=55) |
| Primary Outcome | - Pain in prior month = AIMS2 - Time period of assessment = 9-11 weeks from baseline |
| Findings | - Post-intervention scores, intervention vs control - Pain, mean (SD) = 4.07 (1.99) vs 4.62 (1.79) |
| Limitations | - Lack of control group |
| **Bennell et al, 2017[76]** | |
| Study Design | - Design = RCT - Purpose = evaluate effectiveness of combined exercise and PCST delivered online - Population = chronic knee pain; participated in intervention (n)= 74; lost to follow-up (n)= 4 |
| Intervention | - Modality = website, real-time videoconferencing - Features = education, interactive automated PCST program (PainCOACH), 7 skype sessions with physical therapist - Duration = 12 weeks - Comparator = access to educational material (n=74) |
| Primary Outcome | - Knee pain = 0-10 NRS scale - Function = WOMAC subscale - Time period of assessment = 3 months |
| Findings | - Difference in change between groups, mean change (range) - Pain = 1.6 (0.9-2.3), *P*<0.001 - Function = 9.3 (5.9-2.3), *P*<0.001 |
| Limitations | - Limited external validity as participants had high educational level at baseline and were likely comfortable with using technology; findings may not be generalizable to people with less education or those who are not comfortable with technology |
| **Lawford et al, 2018[85]** | |
| Study Design | - Design = secondary analyses from RCT (Bennell et al) - Purpose = evaluate moderators of the effect of combined exercise and PCST program - Population = knee OA; participated in intervention (n) = 74; lost to follow-up (n) = 9 (at 9 months) |
| Intervention | - Modality = website, real-time videoconferencing - Features = education, interactive automated PCST program (PainCOACH), 7 skype sessions with physical therapist - Duration = 12 weeks - Comparator = access to educational material (n=74) |
| Primary Outcome | - Moderators = age, gender, BMI, education level, employment status, self-efficacy, pain catastrophizing, and expectation of treatment effects - Time period of assessment = 3 and 9 months |
| Findings | - Significant moderators for people who experienced greater reduction in pain at 3 months - Employed adults, between group mean difference (95% CI) = 2.38 (1.52, 3.23) - Self-efficacy, within intervention group reduction in pain per unit increase in self-efficacy (95% CI) = 0.53 (0.28, 0.78) |
| Limitations | - Possible lack of power for some moderators rather than absence of significance given the exploratory nature of the analysis |
| **Mecklenburg et al, 2018[77]** | |
| Study Design | - Design = RCT - Purpose = evaluate efficacy of a digital care program (Hinge Health) for chronic knee pain - Population = chronic knee pain; participated in intervention (n)= 87; lost to follow-up (n)=29 |
| Intervention | - Modality = tablet app, movement trackers, text-messages/emails - Features = 3 sessions of exercise with biofeedback from movement trackers, education, CBT, personal coach, text-messages/email reminders, peer support - Duration = 12 weeks - Comparator = usual care + education on Hinge Health app (n=54) |
| Primary Outcome | - KOOS pain, KOOS-PS - Time period of assessment = 12 weeks |
| Findings | - Between group mean difference (95% CI) - KOOS pain = -7.7 (-12.3, -3) - KOOS-PS = -7.2 (-11.5, -3) |
| Limitations | - High attrition rate |
| **O’Moore et al, 2018[78]** | |
| Study Design | - Design = RCT - Purpose = evaluate efficacy of internet-based CBT - Population = knee OA with MDD; participated in intervention (n)= 44; lost to follow-up (n)=7 |
| Intervention | - Modality = website - Features = online lessons with cartoon narrative teaching CBT skills, regular homework, usual care - Duration = 10 weeks - Comparator = usual care (n=25) |
| Primary Outcome | - Depression symptoms = PHQ-9 - Psychological distress = K-10 - Time period of assessment = 1-week post-intervention (week 11) |
| Findings | - Between group difference, Hedges g (95% CI) - PHQ-9 = 1.01 (0.47, 1.54) - K-10 = 0.75 (0.23, 1.28) |
| Limitations | - Intervention was not tailored for OA management |
| **Stome et al, 2019[81]** | |
| Study Design | - Design = pre/post proof-of-concept study - Purpose = evaluate acceptability, usability, and utility of mobile app for behavior change - Population = OA; participated in intervention (n) = 12; lost to follow-up (n) = 1 |
| Intervention | - Modality = mobile app “Vett” - Features = personal mentor, physical activity/ weight or stress reduction goal achievement plan, self-monitoring, individual feedback, server for progress tracking and communication with mentor, reminders - Duration = 12 week |
| Primary Outcome | - Acceptability = goal achievement on Self-Report Habit Index on a 0-100 scale - Usability = 10 weekly questions on overall satisfaction on a 0-100 scale - Utility = 10 weekly questions on technical feasibility on a 0-100 scale - Time period of assessment = post-intervention (12 weeks) |
| Findings | - Estimated difference from baseline (95% CI) - Acceptability = 22 (17, 26), *P*<0.01 - Usability = 19 (7, 30), *P*=0.04 - Utility = 9 (5, 14), *P*>0.05 |
| Limitations | - Small sample size - Lack of control group |
| **Bennell et al, 2020[82]** | |
| Study Design | - Design = RCT - Purpose = evaluate effect of SMS intervention on adherence to home exercise from TARGET trial - Population = knee OA; participated in intervention (n) = 56; lost to follow-up (n) = 8 |
| Intervention | - Modality = automated text-messaging - Features = 5 weekly text-messages to self-report number of exercise session, ability to report barriers from a pre-determined list for those with ≤2 exercise sessions, behavior change message to help combat barrier selected in previous message - Duration = 24 weeks - Comparator = No SMS (n=54) |
| Primary Outcome | - Adherence = EARS, number of days home exercise completed in past week - Time period of assessment = 24 weeks |
| Findings | - Adjusted mean difference between groups (95% CI) - EARS = 3.1 (0.8, 5.5) - Number of days home exercise completed in past week = 0.6 (0.2, 1.0) |
| Limitations | - No objective outcome measure for adherence - Selection bias as only those participants who completed previous trial (TARGET) were included in analyses |
| **Dharmasari et al, 2020[83]** | |
| Study Design | - Design = mixed-methods analyses from RCT (STAART trial) - Purpose = evaluate feedback from telephone PCST program - Population = African Americans with OA; included in analyses (n) = 93 |
| Intervention | - Modality = telephone - Features = eleven 30-45 min telephone PCST coaching delivered by counselors - Duration = 3 months |
| Primary Outcome | - Overall helpfulness of the PCST program on a 0-10 scale - Thematic analyses of 20 open-ended questions related to experiences with the program - Time period of assessment = post-intervention (3 months) |
| Findings | - Overall helpfulness of program, mean (SD) = 8 (2.2) - Themes related to experience with the program = improved pain coping, mood and emotional benefits, improved physical functioning, experiences related to intervention delivery |
| Limitations | - Limited external validity as many participants were male veterans - Large amount of missing data (i.e., 25% did not complete feedback questions) |
| **Pronk et al, 2020[87]** | |
| Study Design | - Design = RCT - Purpose = evaluate effect of mobile/tablet app on pain and opiate use after KR - Population = post-KR; participated in intervention (n) = 40; lost to follow-up (n) = 2 |
| Intervention | - Modality = mobile/tablet app “PainCoach” - Features = advice on pain medication, exercise, rest, and need for calling the clinic in response to patient’s input of pain experienced - Duration = 2 weeks after surgery - Comparator = usual care (n=36); active use of app, ≥12 total use, (n=19) |
| Primary Outcome | - Knee pain on 0-100 VAS scale - Opiate use - Time period of assessment = 2 weeks after surgery |
| Findings | - Rate of reduction – intervention group vs control, active use of app vs control - Knee pain at rest = 0.3, 1.9 - Knee pain during activity = 1.0, 4.1 - Knee pain at night = 3.0, 6.3 - Opiate use = 23.2, 44.3 |
| Limitations | - Small sample size in active use of app group - Low opiate use among participants at baseline |
| **Buvanendran et al, 2021[86]** | |
| Study Design | - Design = RCT - Purpose = compare effectiveness of CBT delivered via telehealth, in-person, and no CBT - Population = post-KR; participated in 4 week intervention (n)=20 and lost to follow-up (n)=3; participated in 8 week intervention (n)=30 and lost to follow-up (n)=6 |
| Intervention | - Modality = telerehabilitation - Features = CBT lessons and assigned homework - Duration = 4 weeks, 8 weeks - Comparator = in-person CBT (n=15); No CBT (n=15) |
| Primary Outcome | - Change in PCS - Proportion of participants that achieved >4 decrease in WOMAC pain subscale at 3 months - Time period of assessment = 3 months post-op |
| Findings | - Adjusted PCS scores (95% CI) - 4-week telehealth CBT vs no CBT = -5 (-19, 1) - 8-week telehealth CBT vs no CBT = -9 (-15, -1) - 4-week in-person CBT vs no CBT = -5 (-12, -0) - Decrease of >4 in WOMAC pain subscale at 3 months - 4-week telehealth CBT = 29 of 35 (83%) - No CBT = 26 of 33 (79%) |
| Limitations | - Selection bias as participants with chronic opioid use, severe depression, pain catastrophizing, and anxiety were excluded |
| **McCurry et al, 2021[84]** | |
| Study Design | - Design = RCT - Purpose = evaluate effectiveness of CBT delivered by telephone - Population = adults with insomnia and moderate/severe OA; participated in intervention (n) = 139; lost to follow-up (n) = 27 |
| Intervention | - Modality = telephone - Features = six 20-30 min telephone CBT sessions delivered by coaches, sleep diary, education on sleep restriction, stimulus control, sleep hygiene, cognitive restructuring, homework - Duration = 8 weeks - Comparator = education only (n=154) |
| Primary Outcome | - Insomnia = ISI - Time period of assessment = 2 months post treatment, 12 months follow-up |
| Findings | - Adjusted mean between group difference in ISI scores (95% CI) - 2 months post treatment = -3.5 (-4.4, -2.6) - 12 months follow-up = -3 (-4.1, -2) |
| Limitations | - Primary outcome measures did not include OA-symptom related measures - No objective outcome measures included |
| AIMS2 = Arthritis Impact Measurement Scale 2; CBT = Cognitive Behavioral Therapy; CI = Confidence Interval; d = Cohen’s d; EARS = Exercise Adherence Rating Scale; ISI = Insomnia Severity Index; K-10 = Kessler-10; KOOS = The Knee Osteoarthritis Outcome Score; KOOS-PS = KOOS Physical function Short form; MDD = Major Depressive Disorder; NRS = Numeric Rating Scale; OA=Osteoarthritis; PCS = Pain Catastrophizing Scale; PCST=Pain Coping Skills Training; PHQ-9 = 9-Item Patient Health Questionnaire; PT = Physical Therapy; RCT=Randomized Controlled Trial; SMS = Short Messaging Service; VAS = Visual Analog Scale; WOMAC = Western Ontario and McMaster Universities Osteoarthritis Index Score | |

**Table S6: Cost-effectiveness of Digital Health**

| **Cuperus et al, 2016[88]** | |
| --- | --- |
| Study Design | - Design = RCT - Purpose = evaluate cost-effectiveness of a telephone based self-management treatment - Population = generalized OA; participated in intervention (n) = 72 |
| Intervention | - Modality = blended (in-person group sessions, telephone monitoring) - Features = 2 in-person group sessions, 4 telephone sessions delivered by rheumatology nurse and physical therapist, education, goal setting, motivational interviewing, home exercises - Duration = 6 weeks - Comparator = multi-disciplinary in-person group sessions, home exercises (n=75) |
| Primary Outcome | - Self-reported questionnaires regarding medical and non-medical treatment costs - QALY = EQ-5D-3L and SF-6D - Time period of assessment = 6, 26, and 52 weeks |
| Findings | - Total costs €, between group difference - Medical costs = -473, p=0.36 - Societal costs = -708, p=0.75 - QALY Mean difference between groups (95% CI) - EQ-5D-3L = 0.012 (-0.042, 0.066) - SF-6D = 0.022 (0.000, 0.045) |
| Limitations | - Recall bias for self-reported health utilization and cost data - Small sample size |
| **Kloek et al, 2018[92]** | |
| Study Design | - Design = RCT - Purpose = evaluate cost-effectivness of blended PT intervention - Population = knee/hip OA; participated in intervention (n) = 108 |
| Intervention | - Modality = blended (website, in-person PT) - Features = 5 in-person 30-min PT sessions, web-application for graded activity module, exercise, and education - Duration = 12 weeks - Comparator = in-person PT (n=99) |
| Primary Outcome | - Online self-reported questionnaires including intervention, healthcare, sports, informal care, absenteeism, presenteeism, and unpaid productivity costs - QALY = EQ-5D-3L - Time period of assessment = 3 and 12 months |
| Findings | - Mean difference between groups, 95% CI - Intervention costs: -€209 (-294, -128) - Medication costs: -€192 (-436, -79) - Healthcare costs: -€1177 (-3340, 763) - Total costs: -€1371 (-4512, 1240) - Probability of intervention being more cost-effective than comparator QALY = 0.68/0.084 |
| Limitations | - Recall bias for self-reported health utilization and cost data - Sample included both knee and hip OA |
| **Marsh et al, 2014a[89] and 2014b[90]** | |
| Study Design | - Design = RCT - Purpose = evaluate cost-effectiveness of web-based follow-up - Population = post-TJA; participated in intervention (n) = 118 |
| Intervention | - Modality = website, email - Features = radiographs at local facility, online self-report symptom questionnaires, emails to schedule in-person appointment with surgeon if necessary - Duration = 12 months post-op - Comparator = in-person appointment with surgeon and radiographs at clinic (n=111) |
| Primary Outcome | - Proportion of patients who had a significant issue that warranted in-person appointment - Self-reported follow-up costs including transportation, accommodation, healthcare, societal costs - Time period of assessment = 12-months post-op |
| Findings | - Proportion of patients who had a significant issue that warranted in-person appointment = 8 of 25 (32%) - Mean difference between groups (95% CI) - Travel distance = 75.5 (40.92, 110.08) - Travel costs (CAN $) = 10.91 (8.01, 13.01) - Time to complete (min) = 107 (74.89, 139.11) |
| Limitations | - Recall bias for self-reported health utilization and cost data |
| **Tousignant et al, 2015[91]** | |
| Study Design | - Design = RCT - Purpose = compare cost-effectiveness of in-home telerehabilitation and home PT visits - Population = post-KR; participated in intervention (n) = 97 |
| Intervention | - Modality = real-time videoconferencing system - Features = biweekly 45-min sessions delivered by physical therapist - Duration = 8 weeks - Comparator = home PT visits (n=100) |
| Primary Outcome | - Cost analysis from standardized cost sheets during hospital discharge - Time period of assessment = post-intervention (8 weeks) |
| Findings | - Mean difference between groups (95% CI) - Total costs (CAN $) = -263 (-382, -143) - Cost per session = -12.09 (-20.91, -3.27) |
| Limitations | - Need for high-speed internet connection, remote controlled cameras, external speakers and 20-inch LCD screen might not be feasible for people from low SES |
| **Fusco et al, 2016[93]** | |
| Study Design | - Design = Markov decision modelling - Purpose = compare cost-effectiveness of telerehabilitation and in-person PT - Population = post-KR |
| Intervention | - Modality = blended (real-time videoconferencing, in-person PT) - Features = 10 in-person PT sessions, 10 real-time videoconferencing PT sessions delivered by physical therapist - Duration = 3 months after KR - Comparator = 20 in-person PT sessions |
| Primary Outcome | - Incremental costs per QALY = probabilistic sensitivity analysis - Time period of assessment = 3 months after KR |
| Findings | - Probability of cost-effectiveness of digital health - Including transportation = 0.98 - Excluding transportation = 0.56 |
| Limitations | - Assumptions of decision modelling might not reflect clinical practice - Transportation analyses only included ambulance as the mode of transport which might not reflect real-life situations |
| **El Ashmawy et al, 2021[74]** | |
| Study Design | - Design = Retrospective study - Purpose = evaluate cost-effectiveness of virtual follow-up - Population = post-KR/HR; included in analyses (n) = 1749 |
| Intervention | - Modality = VJRC package, telephone calls - Features = VJRC package (instruction letter, PROM booklet, information regarding local radiology for X-Rays), telephone review of PROM and X-Ray outcomes - Duration = in-person consultations at 2-weeks and 6-weeks; virtual follow-up at 1-year, 7-year and every 3 years |
| Primary Outcome | - Cost-analysis = direct and indirect costs for all medical, PT, nursing, radiography, and administrative costs calculated by the Business Finance Department - Time period of assessment = 1 and 7 years after 6-week post-op and then 3-yearly after |
| Findings | - Average appointment cost, VJRC vs in-person consultation (£) = 79 vs 135 - Estimated saving per year (£) = 42, 644 |
| Limitations | - Not directly compared to in-person follow-up |
| CI = Confidence Interval; EQ-5D-3L = EuroQOL 5-domain 3L; HR = Hip Replacement; KR=Knee Replacement; OA=Osteoarthritis; PT=Physical Therapy; QALY = Quality-Adjusted Life Years; RCT=Randomized controlled trial; SES = socio-economic status; SF-6D = Short Form 6D; TJA = Total Joint Arthroplasty; VJRC = Virtual Joint Replacement Clinic | |
